# Plasmatic Profiling of Individuals With Combinations of Type 2 Diabetes Mellitus, Dyslipidemia and Periodontitis: A Cross‐Sectional Study

**DOI:** 10.1111/odi.70313

**Published:** 2026-04-03

**Authors:** François Isnaldo Dias Caldeira, Sâmia Cruz Tfaile Corbi, Alliny de Souza Bastos, Silvana Regina Perez Orrico, Cristiane Ribeiro Salmon, Yizhi Xiao, Walter Luiz Siqueira, Raquel Mantuaneli Scarel‐Caminaga

**Affiliations:** ^1^ Department of Morphology, Genetics, Orthodontics and Pediatric Dentistry, School of Dentistry at Araraquara UNESP‐São Paulo State University Araraquara São Paulo Brazil; ^2^ Department of Diagnosis and Surgery, School of Dentistry at Araraquara UNESP‐São Paulo State University Araraquara São Paulo Brazil; ^3^ Advanced Research Center in Medicine Union of the Colleges of the Great Lakes (UNILAGO) São José do Rio Preto Brazil; ^4^ Dental Research Division, School of Dentistry Paulista University São Paulo São Paulo Brazil; ^5^ College of Dentistry University of Saskatchewan Saskatoon Saskatchewan Canada

**Keywords:** dyslipidemias periodontitis, mass spectrometry, plasma, type 2 diabetes mellitus

## Abstract

**Aim:**

The objective of this study was to investigate the global profile of plasmatic proteins of individuals affected simultaneously or not by type 2 diabetes mellitus (T2DM, well/poorly‐controlled), Dyslipidemia (DL), and Periodontitis (P).

**Methods:**

Besides periodontal examination, plasma was collected for glycemic, and lipid analyses from 150 individuals divided into five groups. The same plasma was submitted to global proteomic investigation using the Liquid Chromatography Electrospray Ionization Tandem Mass Spectrometry (LC‐ESI‐MS/MS), following searching data in the UniProt catalog by Proteome Discoverer 1.3. Funcional enrichment was evaluated by Cytoscape.

**Results:**

In groups (G1)T2DMpoorly‐DL‐P, (G2)T2DMwell‐DL‐P, and (G3)DL‐P, the presence of positively regulated CO4A (mediator of the inflammatory complement cascade) and TTN (sarcomeric protein linked to insulin‐dependent muscle stiffness) proteins was observed, similar to VDBP (vitamin D transporter and systemic inflammatory modulator) protein in groups (G2)T2DMwell‐DL‐P and (G3)DL‐P. Among others, LDLR, DST, SYNE1 (mediators of the lipid homeostasis axis and nuclear‐cytoskeletal integrity) and Ceruloplasmin (involved in iron metabolism and oxidative stress regulation) proteins were exclusively found in respective (G1)T2DMpoorly‐DL‐P, (G2)T2DMwell‐DL‐P, (G3)DL‐P and (G4)Perio groups. Functional enrichment analyses demonstrated complement binding, DNA metabolism and repair among the most up‐regulated PPI.

**Conclusion:**

Each of the combined pathological conditions exhibited a distinct plasmatic proteomic profile. This specificity provides a foundation for future studies to functionally characterize the identified upregulated proteins, both unique and shared. Our findings, therefore, contribute to the identification of potential diagnostic and therapeutic targets for each of these disease scenarios.

## 1 Introduction

Human blood is predominantly utilized in clinical practice for diagnostics and is available in biobanks from thousands of clinical studies (Deutsch et al. 2021). It is a readily obtainable and sensitive sample for determining the health and disease state of individuals and populations, as it contains proteins secreted by various organs (Li et al. 2025). Whole blood, as well as plasma or serum, constitutes a critical circulatory system that houses components of the immune system and is responsible for delivering proteins: in their primary isoforms, sequence variants, post‐translational modifications, and alternative splicing isoforms, to different tissues and organs. Therefore, the interpretation of qualitative, quantitative, and kinetic parameters of these proteins via immunoassays is routinely useful for diagnostics and prognostics, although measuring these plasma and serum proteins is highly challenging and difficult (Deutsch et al. 2021; Li et al. 2025).

Plasma is a biological tissue of extremely complex analysis due to a relatively small number of proteins (approximately 20) that represent 99% of the total plasma protein content, as well as the enormous variability in immunoglobulin sequences. Although studying the profile of proteins present in plasma is challenging, it is fundamental (Li et al. 2025). Several technologies have been employed to measure (Li et al. 2025) and generate qualitative and quantitative data of proteins present in plasma or serum, ranging from assays like the enzyme‐linked immunosorbent assay (ELISA), optimized for the detection of single proteins, to exploratory studies based on mass spectrometry (MS) methodology (Deutsch et al. 2021). MS is commonly used to verify and identify new biomarkers for the clinical monitoring of numerous pathologies such as Alzheimer's disease (Frick et al. 2024), heart disease (Stanley et al. 2004), cancer (Li et al. 2023), metabolic diseases (Cai et al. 2023), periodontitis (Reckelkamm et al. 2023), and type 2 diabetes mellitus (Emilsson et al. 2018).

Diabetes mellitus (DM) is a condition in which environmental and genetic factors play an important role (Yin et al. 2022). It is a chronic disease with a global prevalence (Wang et al. 2021), being estimated that approximately 51% of individuals by 2045 will be affected by DM (Saeedi et al. 2019). The burden of disease caused by type 2 diabetes mellitus (T2DM) is around 90% of the number of people with diabetes worldwide (Saeedi et al. 2019). T2DM is a chronic metabolic disease that manifests persistent and prolonged fasting and postprandial hyperglycemia, which is the main factor associated with the development of complications and comorbidities (Zhao et al. 2022) like stroke, diabetic nephropathy, coronary artery disease, cardiomyopathy, diabetic retinopathy, peripheral arterial disease, and peripheral neuropathy (Athyros et al. 2018).

Recent studies demonstrate that individuals with T2DM are more likely to develop diabetic dyslipidemia (DL) (Athyros et al. 2018). DL can be identified by elevated levels of low‐density lipoprotein cholesterol (LDL‐C), triglycerides (TG), total cholesterol (TC), and reduced high‐density lipoprotein cholesterol (HDL‐C) (Athyros et al. 2018; Gross et al. 2017).

Periodontitis (Perio) is considered a multifactorial condition (Buchwald et al. 2013), and a ubiquitous chronic inflammatory non‐communicable disease with a prevalence of 50% in adults at stages I and II, and of 10% at severe stages III and IV (Watt et al. 2013). While the role of bacteria in the initiation of Perio is primary, host‐related factors, such as immune system reactions, genetic carriage, smoking, and the presence of systemic diseases, including T2DM (Mealey et al. 2006) influence the onset, clinical presentation, and rate of Perio progression (Heitz‐Mayfield 2005).

It is recognized that periodontal disease and T2DM have a two‐way relationship (Taylor 2001), being Perio the sixth largest complication associated with T2DM (Loe 1993). Nowadays, it has been increasingly common to find patients with T2DM who are also affected by the combination of DL and Perio (Nepomuceno et al. 2019). Current studies indicate that these three diseases are intrinsically related as they share common biological mechanisms, are chronic and multifactorial conditions modulated by behavioral, environmental, and genetic factors (Nepomuceno et al. 2019).

However, the specific biological interplay and the unique molecular signatures within this pathological triad remain poorly understood. To our knowledge, no prior studies have investigated the plasma proteome in the context of the simultaneous occurrence of these three pathologies. Here, the hypothesis is that the plasma proteome may be useful for the discovery of new specific biomarkers for diagnosis and clinical monitoring. Therefore, this study aims to analyze the global plasma proteome of individuals with different combinations of well/poorly‐controlled (T2DM, DL), and Perio. We will compare these findings to a healthy Control group and then use functional enrichment and network protein–protein analyses to identify key plasma proteins and better understand their role in each disease combination.

## 2 Materials and Methods

To enhance the quality of the scientific writing of the study, the STROBE (Strengthening the reporting of observational studies in epidemiology) checklist was employed (Cuschieri 2019). The subjects selected for assessing the plasmatic proteome in this study had their circulating lymphocytes and monocytes previously analyzed for transcriptomic data from the same occasion of blood collection (Corbi et al. 2020). This study was approved by the Ethics in Human Research Committee of School of Dentistry at Araraquara (UNESP—São Paulo State University, Araraquara, Brazil; Protocol number 50/06) and was conducted according to the ethical principles of the Declaration of Helsinki.

### 2.1 Study Population and Inclusion Criteria of Biochemical and Periodontal Assessments

Individuals who had never smoked, aged between 35 and 60 years (no matching for sex and age among groups), with at least 15 natural teeth and belonging to a similar demographic context were selected to determine, after a 12‐h overnight fast, their glycemic and lipid profiles through biochemical blood tests. After obtaining the results, they underwent a complete periodontal examination. In addition, each individual underwent a physical examination, including anthropometric data such as abdominal circumference (cm), hip circumference (cm), waist circumference (cm), height (m), weight (kg), and body mass index (BMI). Details regarding the clinical examinations and the inclusion/exclusion criteria for selection of subjects can be found in the (Corbi et al. 2020).

### 2.2 Biochemical Assessments

T2DM participants were referred to the School of Dentistry of Araraquara‐UNESP by specialized endocrinology services, ensuring a profile of established disease under long‐term clinical monitoring (minimum of 2 years), with all diagnoses confirmed by medical records and endocrine evaluation. The participants included in this study were classified as: (i) T2DM subjects who monitored their glycemic control by evaluation of HbA1c, permitting division into poorly controlled subjects (HbA1c ≥ 8.5%, 64 mmol/mol) or well‐controlled subjects (HbA1c < 7.0%) (Corbi et al. 2020). Nondiabetics (normoglycemic individuals) were recruited from the same clinical setting at the School of Dentistry of Araraquara‐UNESP. To ensure their inclusion in the control group, normoglycemia was confirmed through clinical history and recent laboratory records; therefore, nondiabetics were those who presented fasting glucose levels < 100 mg/dL and HbA1c < 5.7%; (ii) for DL, the considered cutoff were the highest values of the National Cholesterol Educational Program (NCEP) Adult Treatment III (ATP III) to avoid individuals with transitory dyslipidemia inclusion: total cholesterol ≥ 240 mg/dL, low density lipoprotein ≥ 160 mg/dL, high density lipoprotein ≤ 40 mg/dL, and triglycerides ≥ 200 mg/dL (National Cholesterol Education Program Expert Panel on Detection, Evaluation, and Treatment of High Blood Cholesterol in Adults 2002).

### 2.3 Periodontal Examination

Complete periodontal examination was performed in six sites per tooth by a single trained calibrated examiner (A.B.S., *Κ* = 0.89), and evaluated periodontal pocket depth (PPD), clinical attachment level (CAL), and bleeding on probing (BOP), using PCP UNC15‐6 (Hu‐Friedy, Chicago, IL, USA), as previously described by Corbi et al. (2020). Diagnosis of Perio was defined by the American Academy of Periodontology and classified individuals III and IV stages, as well as grades A, B and C. Periodontally healthy controls were required to have PPD ≤ 3 mm and BOP in less than 10% of sites (Tonetti et al. 2018).

After considering biochemical and periodontal data, 150 subjects were included in this study and divided into five groups (G) of 30 subjects each: (G1)T2DMpoorly‐DL‐P, composed of individuals with poorly‐controlled T2DM, DL, and Perio; (G2)T2DMwell‐DL‐P, composed of individuals with well‐controlled T2DM, DL, and Perio; (G3)DL‐P, composed of normoglycemic individuals with DL and Perio; (G4)normoglycemic individuals with Perio; and (G5)Control, composed of systemically and periodontally healthy individuals.

### 2.4 Plasma Sample Collection and In–Solution Digestion of Proteins

From the selected subjects, from the same venipuncture to collect blood for biochemical analyses (after a 12‐h overnight fast) and for transcriptome of peripheral blood mononuclear cells (PBMCs), another Vacutainer tube containing heparin was obtained. Each Vacutainer tube was centrifuged at 2000 rpm for 5 min to separate individual plasma (Ventura et al. 2018), which was submitted to depletion of large amounts of albumin and IgG proteins using ProteoPrepTM Immunoaffinity Albumin and IgG Depletion Kit (Sigma‐Aldrich, St Louis, MO, USA). Following this, total protein concentration was measured by the bicinchoninic acid assay (Pierce Chemical, Rockford, IL, USA) using BSA as a protein standard.

An equal 20‐μg protein amount from each subject within the same group was combined to constitute a pooled sample representative of that group (Agho et al. 2021; Ventura et al. 2018; Carvalho et al. 2013; Tilton et al. 2007). These samples were dried in a rotary evaporator, denatured and reduced for 2 h by the addition of 50 μL of 4 M urea, 10 mM dithiothreitol (DTT), and 50 mM NH_4_HCO_3_, pH 7.8, and incubation for 1 h at room temperature. The solution was diluted with the addition of 150 μL of 50 mM NH_4_HCO_3_, pH 7.8. After addition of 2% w/w sequencing‐grade trypsin (Promega, Madison, WI, USA), tryptic digestion was carried out for at least 16 h at 37°C in a water bath. Finally, samples were dried in a rotary evaporator, de‐salted by C‐18 ZipTip1Pipette Tips (Millipore, Billerica, MA, USA), and subjected to mass spectrometry (Siqueira and Dawes 2011).

### 2.5 Liquid Chromatography Electrospray Ionization Tandem Mass Spectrometry (LC‐ESI‐MS/MS)

After trypsinization, equal amount of all the samples was resuspended in 15 μL of 97.5% H_2_O/2.4% acetonitrile/0.1% formic acid and then subjected to reversed phase LC‐ESI‐MS/MS, using LTQ‐Velos (Thermo Scientific, San Jose, CA, USA) mass spectrometer. Liquid chromatography separation was achieved using a C18 column of capillary fused silica (column length 10 mm, column ID 75 μm, 3‐μm spherical beads, and 100 Å pore size) linked to the mass spectrometer through electrospray ionization. Peptides were eluted from the nanoflow reversed‐phase HPLC over a linear 85 min gradient ranging from 5% to 55% of solvent B (97.5% acetonitrile, 0.1% formic acid), at a flow rate of 300 nL/min, with a maximum pressure of 280 bar. Electrospray voltage and the temperature of the ion‐transfer capillary were 1.8 kV and 250°C, respectively. The survey scan was set in the range of *m*/*z* values 390–2000 MS/MS. Each survey scan (MS) was followed by automated sequential selection of seven peptides for a standard collision‐induced CID, with dynamic exclusion of the previously selected ions (Siqueira and Dawes 2011).

### 2.6 Protein Identification

The acquired MS/MS spectra were searched against Human protein databases (Swiss PROT and TrEMBL, Swiss Institute of Bioinformatics, Geneva, Switzerland, http://ca.expasy.org/sprot/) using Proteome Discoverer 1.3 software (Thermo Scientific, San Jose, CA, USA) and SEQUEST algorithm. The search parameters using SEQUEST included: 1.5, 2.5, 3.1, 3.1, 4.5. Parameter Xcorr was used. A filter was applied to the peptide sequences during the import that eliminated all sequences with a Percolator *q*‐value greater than 1% (false discovery rate). Peptides were grouped into proteins, and a protein ratio and *p*‐value were calculated (Siqueira and Dawes 2011). A total of 5 runs per group were carried out in the mass spectrometer. For positive identification, the same protein had to be identified in at least three runs from the same group.

### 2.7 Bioinformatics

Resulting quantitative values (spectrum counts, PSM) were used to analyze the distribution of identified proteins in each group of subjects. Only proteins identified with at least two unique peptides were considered in the results. Principal Component Analysis (PCA) of each studied group was performed on raw values converted to the log_2_FC. The R statistical software function “prcomp” then determined the principal component values, with graphical representation provided by the “ggfortify” package (from “ggplot2”). The Heatmap graphic organization was constructed by converting NSC into z‐scores and submitting them to the “pheatmap” package.

Proteins identified in each group were compared using the Venny 2.0 online tool (Oliveros 2015). An interactive tool for comparing lists with Venn's diagrams (http://bioinfogp.cnb.csic.es/tools/venny/index.html) consulted on 2025‐06‐16. log_2_FC (log_2_FC ≥ ±10.0 and log_2_FC ≤ −10) of each diseased (G1, G2, G3, G4) group compared with the Control (G5) group was determined (i.e., G1 versus G5; G2 versus G5; G3 versus G5; G4 versus G5) to obtain lists of up/down‐regulated proteins (log_2_FC), as well to perform enrichment analyses.

Pathway and enrichment analyses of up‐regulated differentially abundant proteins of each group comparison were carried out using the Metascape.org platform (Zhou et al. 2019) with the following ontology sources: Gene ontology (GO) Biological Processes, PANTHER Pathway, KEGG Pathway, Reactome Gene Sets, WikiPathways, Canonical Pathways, and CORUM. All genes in the genome have been used as an enrichment background. Terms with a *p*‐value < 0.01, a minimum count of 3, and an enrichment factor > 1.5 were collected and grouped into clusters based on their membership similarities. The enrichment factor is the ratio between the observed counts and the counts expected by chance. More specifically, *p*‐values were calculated based on the cumulative hypergeometric distribution, and *q*‐values were calculated using the Benjamini‐Hochberg procedure to account for multiple testing.

From protein–protein interaction networks were constructed using STRING‐db (Szklarczyk et al. 2023), the up‐regulated differentially abundant proteins of each group comparison were submitted to Cytoscape (3.10.1) (Shannon et al. 2003) to visualize the proteins network plot. The minimum required interaction score was set to medium confidence (0.400), and disconnected nodes were hidden. Network statistics including number of nodes, edges, average node degree, clustering coefficient, and PPI enrichment *p*‐value were recorded. MCL (Markov Cluster Algorithm) clustering was applied with an inflation parameter of 1.5 to identify functional modules (Brohee and van Helden 2006). Clusters were annotated based on STRING functional enrichment. Data consulted on 2025‐06‐20.

The PANTHER GO‐Slim (PANTHER version 19.0, consulted on 2025‐06‐25, (http://www.pantherdb.org/)) was utilized for enrichment analysis of differentially abundant proteins in each group comparison, analyzed according to Molecular Function and Protein Class terms. To enable qualitative comparison among groups, the number and abundance of proteins participating in specific pathways within each comparative group were quantitatively represented by a bar graph, detailing the proteins identified in each enriched term.

### 2.8 Statistical Analysis

The distribution and normality of the characteristics of each group were evaluated by the D'Agostino‐Pearson test. The Chi‐squared test (for gender) and Kruskal‐Wallis test, followed by Dunn's post‐test, was used to compare other sample characteristics, by using GraphPad Prism software, version 5.0. Differentially expressed proteins used for pathway enrichment analysis with Metascape were defined as those with *p* < 0.05 in this study. Proteome data were analyzed using R software (with RStudio extension) and followed by Student's *t*‐test (*p* < 0.05). The fold‐change was calculated as the ratio of G1, G2, G3, G4/G5 values.

## 3 Results

### 3.1 Clinical Parameters

Subject characteristics are presented in Table 1 as the median and 25th–75th interquartile range, to better represent the data distribution, since some of them were previously presented by Corbi et al. (2020). There were no significant differences in gender among groups. Regarding biochemical findings, independently of the poor or well‐controlled HbA1c levels, T2DM groups had significantly higher levels of fasting glucose, HbA1c, and HOMA‐IR index, without difference between them regarding insulin levels and insulin resistance index (HOMA‐IR) (Table 1). Diabetic groups (T2DMpoorly‐DL‐P and T2DMwell‐DL‐P) demonstrated similar overweight when compared with (G5)Control, as well as similar very high risk for cardiovascular disease development given by the high waist‐to‐hip ratio and abdominal circumference values (“Obesity: preventing and managing the global epidemic. Report of a WHO consultation,” 2000) (Table 1). DL was well characterized in subjects from the first three groups, as demonstrated by higher levels of total cholesterol, LDL, and triglycerides. Periodontal tissue destruction and local inflammation, as denoted by high index of marginal bleeding, probing depth, clinical attachment loss, and percentage of periodontal sites with suppuration, were significantly more severe in diabetics, particularly in (G1)T2DMpoorly‐DL‐P (Table 1).

**TABLE 1:** Characteristics of the studied population: Demographic, physical, biochemical, and periodontal data.

Variable	(G1)T2DMpoorly‐DL‐P	(G2) T2DMwell‐DL‐P	(G3)DL‐P	(G4)Perio	(G5)Control
Median (25th–75th IQR)	*n* = 30	*n* = 30	*n* = 30	*n* = 30	*n* = 30
Gender (M/F)^#^	12/18	10/20	13/17	11/19	12/18
Age	48.5 (41.2–55)^a^	52 (46.5–56)^a^	51 (43–55)^a^	45 (40.5–47)^a^	40.5 (36–42)^b^
BMI (kg/m^2^)*	28.9 (26.8–34.4)^a,c^	30.5 (29.1–33.3)^c^	28.0 (26.1–30.9)^a,c^	26.2 (23.7–29.3)^a,b^	23.7 (22.2–27.5)^b^
Waist‐to‐hip ratio*	1.0 (0.9–1.0)^a,b^	1.0 (0.9–1.0)^b^	0.9 (0.9–1.0)^a,b^	0.9 (0.9–1.0)^a^	0.8 (0.8–0.9)^c^
Abdominal circumference (cm)*	101.5 (97.0–111.8)^a,b^	110.0 (102.0–117.0)^b^	98.0 (93.0–106.0)^a,c^	96.0 (92.0–105.0)^a,c^	85.5 (79.8–97.5)^c^
Hs C‐reactive protein (mg/dL)*	0.2 (0.1–0.7)^a,b^	0.5 (0.2–1.1)^b^	0.2 (0.1–0.5)^a,b^	0.2 (0.0–0.4)^a^	0.1 (0.0–0.3)^a^
Fasting glucose (mg/dL)	209.5 (169.5–290.0)^a^	125.0 (111.0–162.0)^a^	89.0 (86.0–95.0)^b^	91.0 (86.0–99.0)^b^	86.0 (80.0–92.0)^b^
Insulin (U/L)	13.1 (8.8–17.6)^a,c^	13.9 (9.8–19.9)^c^	11.0 (5.4–19.9)^a,b,c^	7.7 (5.3–10.7)^a,b^	6.5 (4.7–7.8)^c^
HbA_1c_ (%)	9.7 (8.9–11.4)^a^	6.5 (6.2–6.7)^b^	5.5 (4.7–5.9)^c^	5.1 (4.7–5.4)^c^	5.4 (5.3–5.5)^c^
HOMA‐IR	6.4 (4.5–10.0)^a^	5.2 (3.0–7.6)^a^	2.2 (1.2–4.4)^b^	1.6 (1.2–2.7)^b^	1.3 (1.0–1.6)^b^
Total cholesterol (mg/dL)	244.0 (225.3–266.3)^a^	253.0 (201.0–271.0)^a^	249.0 (220.0–264.0)^a^	179.0 (161.0–190.0)^b^	181.5 (163.3–197.0)^b^
HDL cholesterol (mg/dL)	41.5 (39.0–51.3)^a^	43.0 (40.0–53.0)^a^	49.0 (43.0–58.0)^a^	47.0 (41.0–51.0)^a^	48.5 (44.0–53.0)^a^
Non‐HDL‐C	192.0 (177.3–220.5)^a^	203.0 (161.0–222.0)^a^	195.0 (172.0–210.0)^a^	121.0 (110.0–142.0)^a^	132.5 (115.0–144.3)^a^
LDL cholesterol (mg/dL)	160.1 (130.3–174.3)^a^	151.0 (109.0–184.2)^a^	162.6 (134.8–180.2)^a^	102.2 (92.2–116.4)^b^	113.9 (98.6–126.8)^b^
Triglycerides (mg/dL)	219.0 (137.8–270.3)^a^	253.0 (206.0–308.0)^a^	200.0 (134.0–231.0)^a^	89.0 (65.0–126.0)^b^	85.0 (64.3–105.3)^b^
Percentage of sites with visible plaque	79.9 (68.2–88.3)^a^	72.1 (64.6–78.7)^a^	69.0 (62.0–79.8)^a^	53.8 (47.9–75.0)^a^	16.1 (9.8–20.0)^b^
Percentage of sites with marginal bleeding	60.5 (48.1–70.2)^a^	45.0 (36.3–58.7)^a,b^	35.9 (30.0–46.6)^a^	40.2 (32.6–50.0)^a^	8.0 (5.4–11.5)^c^
Percentage of sites with bleeding on probing	69.2 (58.3–80.7)^a^	56.7 (44.7–64.1)^b^	51.2 (44.0–59.1)^b^	53.8 (46.3–60.7)^b^	12.7 (10.0–16.8)^c^
Number of teeth	22.0 (19.8–26.0)^a^	21.0 (18.0–26.0)^a^	24.0 (21.0–26.0)^a^	25.0 (22.0–27.0)^a^	28.0 (27.0–28.0) ^b^
Percentage of sites with probing depth ≤ 3 mm	46.0 (32.8–52.0)^a^	61.0 (47.0–66.0)^a,c^	65.0 (56.0–73.0)^c^	48.0 (44.0–60.0)^a,c^	99.0 (98.8–100.0)^b^
Percentage of sites with probing depth = 4–5 mm	32.0 (22.8–38.0)^a,b^	30.0 (25.0–37.0)^b^	28.0 (24.0–39.0)^b^	42.0 (38.0–48.0)^a^	1.0 (0.0–1.3)^c^
Percentage of sites with probing depth ≥ 6 mm	26.5 (8.8–37.3)^a^	11.0 (4.0–18.0)^a,b^	5.0 (2.0–9.0)^b^	4.0 (2.0–10.0)^b^	0.0 (0.0–0.0)^c^
Percentage of sites with attachment loss ≤ 2 mm	12.5 (7.8–15.0)^a,c^	16.0 (4.0–20.0)^a,c^	24.0 (15.0–33.0)^a^	4.0 (1.0–17.0)^c^	63.5 (58.0–74.0)^b^
Percentage of sites with attachment loss = 3–4 mm	37.5 (26.8–46.0)^a,b^	48.0 (37.0–57.0)^a^	52.0 (44.0–58.0)^a,c^	62.0 (55.0–69.0)^c^	36.5 (26.0–42.0)^b^
Percentage of sites with attachment loss ≥ 5 mm	47.0 (40.5–56.3)^a^	31.0 (22.0–46.0)^a,b^	21.0 (15.0–30.0)^b^	29.0 (22.0–36.0)^b^	0.0 (0.0–0.0)^c^
Number of sites with suppuration	5.5 (2.0–12.5)^a^	1.0 (0.0–4.0)^a,b^	1.0 (0.0–1.0)^b^	0.0 (0.0–2.0)^b^	0.0 (0.0–0.0)^c^

*Note:* The Kruskal–Wallis' test (*p < 0.05) was performed, followed by Dunn's post‐test. Different letters ^abc^ mean significant differences among groups.

Abbreviations: Hs C‐reactive protein, high sensitive C‐reactive protein; IQR, interquartile range.

^#^ Males/females' similar distribution among groups (*p* = 0.931), Chi‐squared test.

* Cardiovascular risk factors.

### 3.2 Plasmatic Proteomic Profile Analyses

Plasmatic proteome from all five different groups showed a consistent elution of protein/peptides range from 10 to 60 min (Figure S1). Moreover, albumin and IgG were efficiently depleted from the plasma samples (Figure S2). The plasmatic proteomic profile of each group is presented in Figure 1. PCA, performed to transform and organize dataset of each group in an unsupervised method, presented first principal component (PC1) = 43.79% of the variance, whereas the second principal component (PC2) showed 28.09% (Figure 1A). In other words, the samples with similar protein profiles were clustered together, leading to three different clusters of the five groups, showing a distinction between the plasmatic proteome of the (G5)Control; the (G4)Perio; and the groups affected by simultaneous comorbidities (G1, G2, and G3), being (G2)T2DMwell‐DL‐P and (G3)DL‐P more similar to each other (Figure 1A). Figure 1C presented hierarchical clustering of the top 50 differentially abundant proteins in each group, being the highest distinction between (G5)Control and (G1)T2DMpoorly‐DL‐P, which was different from G2; while G3 and G4 had more similar proteomic profiles in the heatmap analysis. In Figure 1B the Venn diagram presented the distribution of the total (1542) proteins, while the 10 exclusive most abundant proteins in each group are presented in Table S5, besides the 61 (2.8%) of common proteins to all five groups shown in Table S6. Figure 1D shows the Venn diagram of differentially expressed proteins (up‐ and down‐regulated). A total of 980 proteins were observed in relation to positively regulated proteins, while negatively regulated proteins showed a total distribution of 16 proteins in the five groups investigated. From the list of proteins and the respective PSM values of each group comparison, we ranked the 25 most up‐regulated proteins (with the highest log_2_FC) and the 25 most down‐regulated proteins (with the lowest log_2_FC) (Tables S1–S4).

**FIGURE 1:**
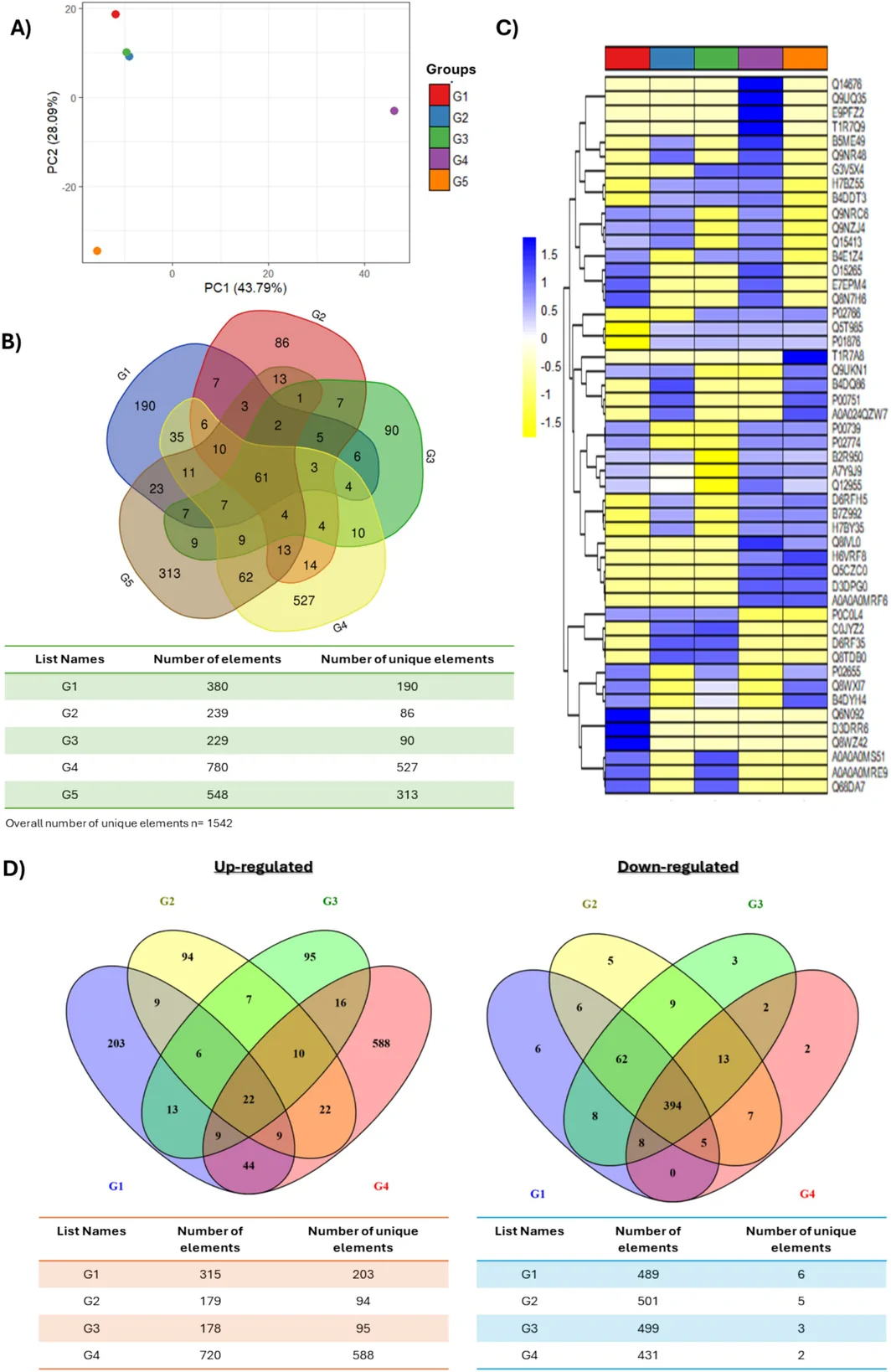
Proteomic profile of (G1)T2DMpoorly‐DL‐P, (G2)T2DMwell‐DL‐P, (G3)‐DL‐P, (G4)Perio, and (G5)Control groups.

### 3.3 Specific Proteins

In the (G1)T2DMpoorly‐DL‐P versus (G5)Control, the most abundant protein was Complement C4‐A (CO4A, log_2_FC = 6.73, Table S1), followed by Complement C2 (B4E1ZE, log_2_FC = 6.11), both essential proteins in the classical pathway of the complement system, which culminate in pathogens elimination. Thirdly, the TITIN (TTN) protein has the ATP binding or protein kinase activity as the molecular function (log_2_FC = 5.7). Among the exclusive proteins in (G1)T2DMpoorly‐DL‐P (Table S5), we highlight the Inter‐alpha‐trypsin inhibitor heavy chain 2 (ITIH2) and the Low‐density lipoprotein receptor (LDLR). Figure 2A showed functional enrichment analysis of the up‐regulated plasmatic proteins identified in (G1)T2DMpoorly‐DL‐P, in a magnitude degree of richness in biologically connected pathways and processes. Main enrichment findings of Metascape were “Growth” (GO:0040007; Log_10_(*q*) −1.79), “DNA biosynthetic process” (GO:0071897; Log_10_(*q*) −1.79); “Chromosome organization” (GO:0051276); and “Regulation of Complement cascade” (R‐HAS‐977606; Log_10_(*q*) −1.79). Figure 4A presented a network with a higher magnitude in richness of protein–protein connections (PPI) clustered using MCL. STRING‐db network clustering highlighted key functional subgroups, particularly those associated with “DNA repair”, with 19 positively regulated proteins in this clustered MLC association (PPI enrichment *p*‐value: < 1.0e^−16^). The most significantly enriched clusters included “Methyltransferase activity”, with 17 positively regulated proteins in this clustered MLC association (PPI enrichment *p*‐value: < 1.0e^−16^) (Figure 4A).

**FIGURE 2:**
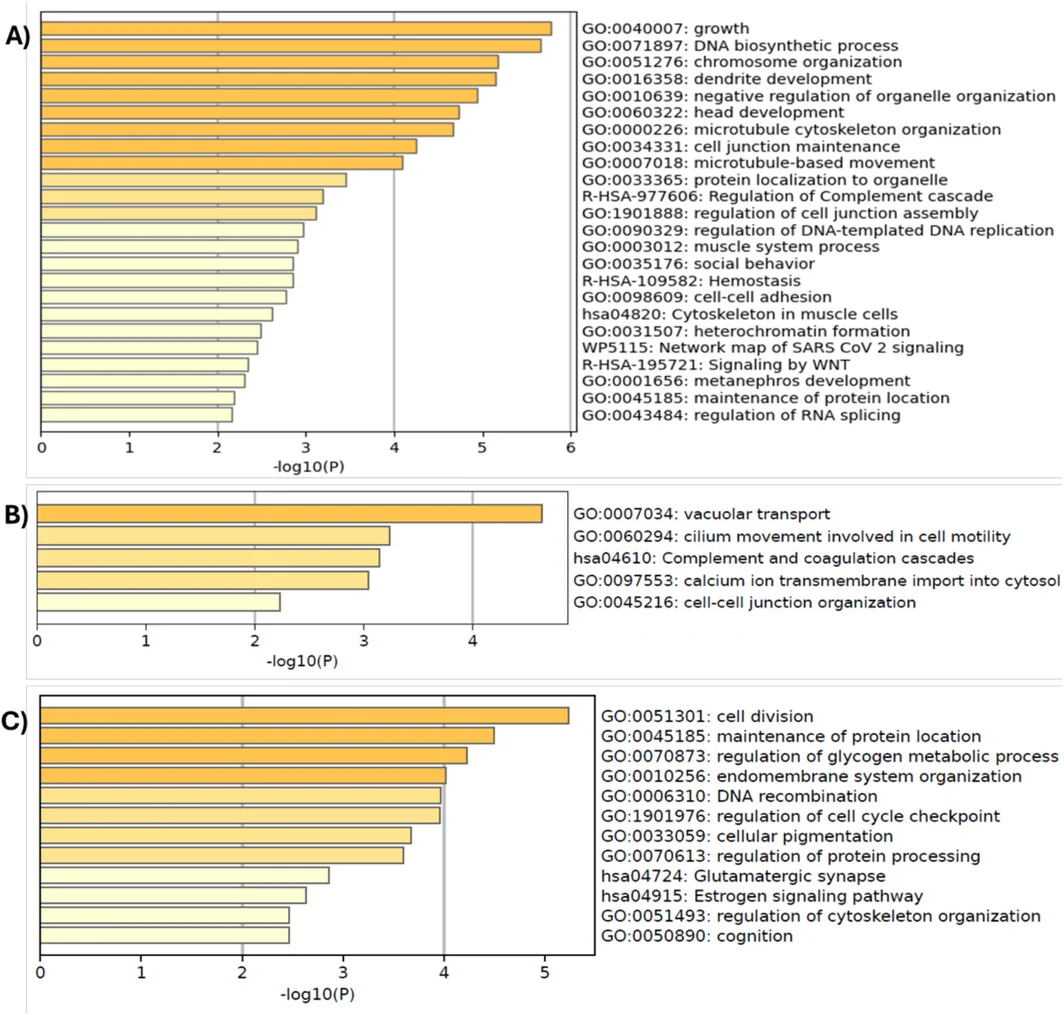
Pathway and process enrichment analyses of up‐regulated differentially abundant proteins of diseased groups compared with Control group. (A) Enrichment analyses of (G1)‐T2DMpoorly‐DL‐P. (B) Enrichment analyses of (G2)‐T2DMwell‐DL‐P. (C) Enrichment analyses of (G3)‐DL‐P. Enrichment analyses carried out with GO Biological Processes, PANTHER Pathway, KEGG Pathway, Reactome Gene Sets, WikiPathways, Canonical Pathways, and CORUM.

In metabolic compensated diabetic subjects (G2)T2DMwell‐DL‐P versus (G5)Control the most abundant protein found was also CO4A (log_2_FC = 6.99, Table S2), followed by Vitamin D‐binding protein (D6RF35, log_2_FC = 6.09) involved in vitamin D transport and storage. Again TTN (log_2_FC = 4.75) was a higher expressed protein. In Figure 2B, a less complex network of biologically connected pathways and processes was found in comparison with the last mentioned (G1)T2DMpoorly‐DL‐P group. The main enrichment findings by Metascape are “Complement and coagulation cascades” (hsa04610; Log_10_(*q*) 0.00), “Calcium ion transmembrane import into cytosol” (GO:0097553; Log_10_(*q*) 0.00) and “Cell‐cell junction organization” (GO:0045216: Log_10_(*q*) 0.00). Figure 4B presented also a less complex network of PPI connections, in which STRING‐db network cluster highlight key functional subgroups, particularly “Calcium channel”, with six positively regulated proteins in this clustered MLC association (PPI enrichment *p*‐value: < 2.67e^−07^). Following there is the “Complement binding”, with three proteins (GC: Vitamin D‐binding protein; CO4A: Complement C4‐A; C8G: Complement component C8 gamma chain/PPI enrichment *p*‐value: 0.000338).

Up‐regulated proteins in (G3)DL‐P in comparison with (G5)Control showed again CO4A as the high abundant protein (log_2_FC = 6.94, Table S3), as found in the (G1)T2DMpoorly‐DL‐P and (G2)T2DMwell‐DL‐P groups. This was followed by Complement C2, Vitamin D‐binding protein (VDBP, encoded by the *GC* gene, UNIPROT D6RF35, log_2_FC = 6.04), and Gelsolin (encoded by the *GSN* gene, UNIPROT A0A0A0MS51, log_2_FC = 5.32), which is responsible for calcium‐regulated, actin‐modulating protein that binds to the plus (or barbed) ends of actin monomers. Additionally, TTN was also found with log_2_FC = 5.32. This group presented in Figure 2C, among main enrichment functions accessed by Metascape: “Regulation of glycogen metabolic process” (GO:0070873; Log_10_(*q*) −0.46), “DNA recombination” (GO:0006310; Log_10_(*q*) −0.46), “Regulation of cell cycle checkpoint” (GO:1901976; Log_10_(*q*) −0.46), and “regulation of protein processing” (GO:0070613; Log_10_(*q*) −0.22). Figure 5A showed the network of PPI connections, in which STRING‐db network clustering highlighted “Positive regulation of protein processing in phagocytic vesicles”, with eight positively regulated proteins (among them GC, GSN, and C4A; PPI enrichment *p*‐value: 3.62e^−08^). The following most significantly enriched cluster was the “Regulation of glycogen metabolic process”, with eight proteins (among them IRS1; PPI enrichment *p*‐value: 8.59e^−05^).

For the (G4)Perio versus (G5)Control group comparison, the highest abundance was found for the Mucin‐16 (MUC16; log_2_FC = 7.09), Ceruloplasmin (encoded by the *CP* gene, UNIPROT E9PFZ2; log_2_FC = 7.0), and the exclusive SRRM2 (Serine/arginine repetitive matrix protein 2; log_2_FC = 5.46, Tables S4 and S5). This agrees with the Metascape functional enrichment analysis (Figure 3), for the terms “Regulation of protein‐containing complex assembly” log(*q*) = −6.05 (GO:0043254), “DNA metabolic process” log(*q*) = −2.50 (GO:0006259), “Regulation of DNA metabolic process” log(*q*) = −2.42 (GO:0051052) and “Lysine degradation” log(*q*) = −1.86 (hsa00310). This group, shown in Figure 3, has a magnitude degree of richness in biologically connected pathways and processes (*x* axis). Figure 5B showed STRING‐db network clustering highlighted key functional subgroups, particularly those associated with “Structural constituent of cytoskeleton”, with Tenascin‐C (encoded by the *TNC* gene, log_2_FC = 4.64), one of the 56 proteins composed of clustered MLC association (PPI enrichment *p*‐value: < 1.0e^−16^). Finally, the most significantly enriched cluster was “DNA repair”, with 34 proteins, one of them the exclusive Mediator of DNA damage checkpoint protein 1 (MDC1), clustered by MLC association (PPI enrichment *p*‐value: < 1.0e^−16^) (Figure 5B).

**FIGURE 3:**
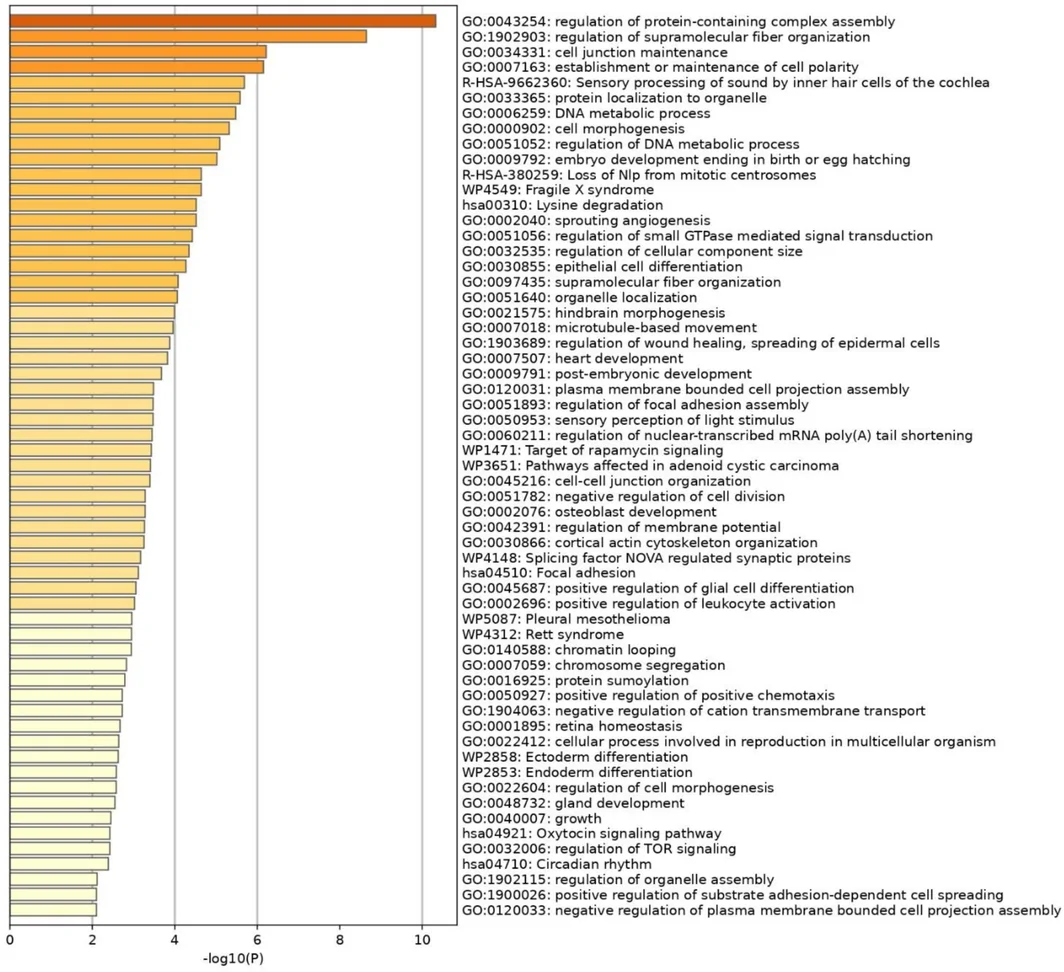
Pathway and process enrichment analyses of up‐regulated differentially abundant proteins of Periodontitis compared with Control group. Enrichment analyses carried out with GO Biological Processes, PANTHER Pathway, KEGG Pathway, Reactome Gene Sets, WikiPathways, Canonical Pathways, and CORUM.

GO enrichment analyses were performed in PANTHER GO‐Slim for the additional functional interpretation of the up regulated proteins with differential abundance in each diseased group compared to the control group, according to Molecular Function and Protein Class terms (Figure S3).

## 4 Discussion

To our knowledge, this is the first study to perform a global proteomic analysis of plasma in patients who simultaneously presented a combination of T2DM, DL, and Perio. This comprehensive proteomic study provides important insights into the protein levels, metabolic pathways, and molecular mechanisms shared by these three complex diseases of high global prevalence. As the groups showed a similar distribution of men and women (*p* = 0.931), and although the Control group was significantly younger than the other groups, participants were predominantly within the third to sixth decades of life. Therefore, we consider that potential differences related to sex and age had a limited impact on the interpretation of the plasma proteomic profile (including protein concentrations and lipid‐related components). Plasma of these different pathological conditions was used in an attempt to find biomarkers for each clinical diagnosis which could be easily assessed. The main findings obtained through unsupervised PCA and hierarchical clustering analyses highlight similarities in the plasmatic proteomic profile between individuals from the (G2)T2DMwell‐DL‐P and those from the (G3)DL‐P groups. These groups were distinct from the (G1)T2DMpoorly‐DL‐P, and much more from the group with isolated periodontitis (G4)Perio. All diseased groups demonstrated a plasmatic proteomic profile distinct from the (G5)Control group. It is possible to note that the poor metabolic control of T2DM, a systemic disease, significantly impacts the plasmatic proteomic profile, just as inflammation caused by periodontitis, a condition localized in the oral cavity, also influences distinct circulating systemic protein expression levels. Moreover, the biochemical and periodontal data in Table 1 show that the (G1)T2DMpoorly‐DL‐P group exhibited the worst parameters, making it the most distinct from the (G5)Control group, which aligns with the unsupervised PCA findings.

In regard to the plasmatic proteome profiling of (G1)T2DMpoorly‐DL‐P group, we identified a significant increase in the abundance of CO4A and Complement C2, and to the best of our knowledge from a comprehensive literature review, this is the first study to report elevated levels of these proteins in plasma of individuals with T2DM‐DL‐P (both poorly‐ and well‐controlled), as well as with isolated DL‐P. Both CO4A and Complement C2 are important precursor proteins of the classical complement pathway that work together in a sequence of events responsible for acting in the defense against pathogens and strengthening the adaptive immune system. Current evidence suggests that the activation of the complement system cascade is implicated in the etiopathogenesis of several inflammatory diseases, such as rheumatoid arthritis, cardiovascular diseases, asthma, and T2DM (Fujita et al. 2013). As demonstrated by Fujita et al. (2013), patients with elevated plasma levels of C3 and C4 proteins were correlated with abnormalities in glucose and lipid metabolism. Furthermore, the expression levels of complement activation products, especially the C4 protein, were almost twice as high in individuals with T2DM compared to normoglycemic individuals, demonstrating that the complement pathway is activated in T2DM patients (Fujita et al. 2013). In agreement, Muscari and collaborators (Muscari et al. 1995) reported that plasma levels of C3 and C4 were correlated with triglyceride and total cholesterol levels, which are considered important risk factors for the development of dyslipidemia and the risk of acute myocardial infarction. Therefore, considering that CO4A is an important protein associated with inflammatory diseases, we hypothesize that it could act as a key biomarker for detecting a dyslipidemic state, given its high score value in the (G1)T2DMpoorly‐DL‐P (log_2_FC = 6.73), (G2)T2DMwell‐DL‐P (log_2_FC = 6.99), and (G3)DL‐P (log_2_FC = 6.94) groups. However, it is of utmost importance that these findings be validated in other populations and with a larger patient cohort. Among the proteins exclusive to the (G1)T2DMpoorly‐DL‐P group (Table S5), we highlight the Inter‐alpha‐trypsin inhibitor heavy chain 2 (ITIH2). This protein is involved in pathways related to the regulation of insulin‐like growth factor (IGF), its transport and uptake by insulin‐like growth factor binding proteins (IGFBPs), and protein metabolism (mês). This finding is consistent with a systematic review of circulating proteins in type 1 diabetes (T1DM), which reported both ITIH2 and Complement C4‐A (CO4A) in at least two independent studies. Interestingly, that review found that T1DM circulating biomarkers were associated with pathways related to complement, lipid metabolism, and immune response, which is a profile broadly similar to the plasmatic proteome of the T2DM individuals in our study (Sarkar et al. 2023). Moreover, the low‐density lipoprotein receptor (LDLR) is another exclusive protein found in (G1)T2DMpoorly‐DL‐P (Table S5). The rise in LDLR increases LDL particle uptake by pancreatic cells, thereby promoting toxicity of cholesterol on the β‐cells, its dysfunction, and apoptosis, especially in cells with baseline glucose disturbances from DM subjects. On the contrary, reduced LDLR amount, or function observed in hypercholesterolemia familiar would protect the cells against LDL particle entry, whereas their increase would promote it and, thus, damage the pancreatic β‐cells (Gonzalez‐Lleo et al. 2022). The (G1)T2DMpoorly‐DL‐P group also uniquely expressed Ras‐responsive element‐binding protein 1 (RREB1) and Lysine‐specific demethylase 7A (KDM7A), which showed significant enrichment in the “Methyltransferase activity” pathway (Figure 4A). This finding, combined with the enrichment of key “DNA repair” proteins like BRCA2, TP53BP1, and RAD50, suggests that the observed protein–protein interaction (PPI) patterns are consequences of the poor glycemic control, obesity, dyslipidemia, and more severe periodontitis present in this patient cohort. These findings may help elucidate the biological mechanisms underlying the increased risk of liver, pancreatic, endometrial, colorectal, bladder, and breast cancers observed in individuals with T2DM (Suh and Kim 2011).

**FIGURE 4:**
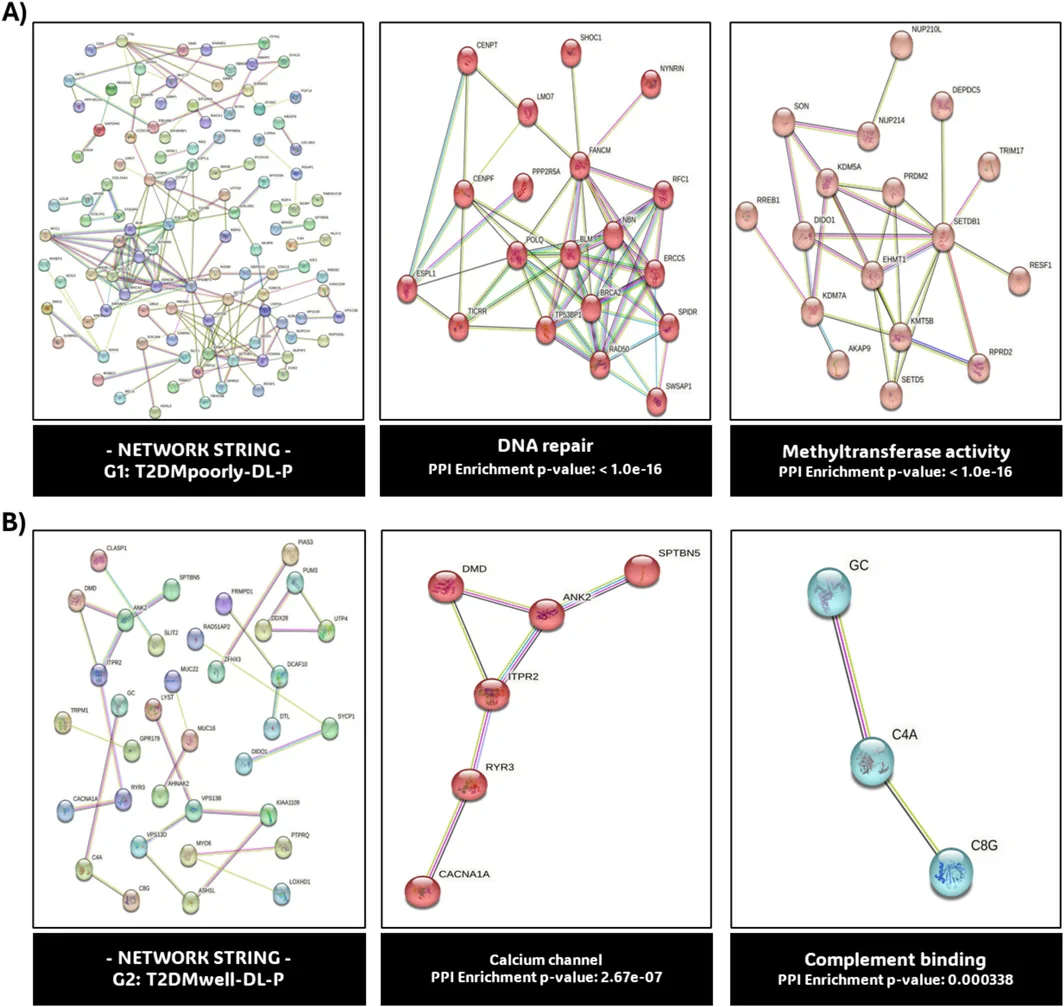
STRING‐db‐based visualization of the protein–protein interaction (PPI) network clustered using the Markov Cluster Algorithm (MCL). (A) Protein–Protein interaction enrichment analysis of up‐regulated (G1)‐T2DMpoorly‐DL‐P. (B) Protein–Protein interaction enrichment analysis of up‐regulated (G2)‐T2DMwell‐DL‐P. PPI network clustered using the MCL clustering. Inflation parameter: 1,5. For a more detailed view of each protein individually, including protein names, please refer to the Figures S4–S9.

Vitamin D‐binding protein (VDBP) was found up‐regulated in the (G2)T2DMwell‐DL‐P and (G3)DL‐P groups. This protein is involved in the transport and storage of vitamin D, scavenging of extracellular G‐actin, enhancement of the chemotactic activity of C5 alpha for neutrophils in inflammation and macrophage activation (GeneCards‐VDR 2025). The study by Turkes et al. (2021) showed significant positive correlations between C‐reactive protein (CRP) and VDBP levels in T2DM individuals. The overproduction of pro‐thrombotic factors, monocyte migration and activation, and the up‐regulation of endothelial pro‐inflammatory cytokines are recognized mechanisms that increase VDBP production (Joshi et al. 2016; Turkes et al. 2021). Growing epidemiological evidence revealed a strong relationship between reduced circulating vitamin D levels, remnant cholesterol levels, and the lipid profile (Afzal et al. 2013; Feng et al. 2020). Therefore, since T2DM individuals demonstrate dyslipidemia and higher CRP (Turkes et al. 2021), it is expected that the VDBP protein levels would also be increased. Our findings endorse this association, as the VDBP protein levels in (G2)T2DMwell‐DL‐P and (G3)DL‐P groups were similarly higher and agreed with dyslipidemia and higher CRP; also in agreement with PCA and hierarchical clustering analyses. Dystonin (DST) protein was exclusively found here in the (G2)T2DMwell‐DL‐P group, and a meta‐analysis demonstrated that DST (Dystonin/BPAG1) was identified upregulated in two diabetic mouse models that focused on synaptic adhesion proteins. The authors concluded that DST alteration may be involved in the mechanism of diabetic dementia (Cheng et al. 2019).

We found TTN among the first five most abundant up‐regulated proteins in (G1)T2DMpoorly‐DL‐P, (G2)T2DMwell‐DL‐P, and (G3)DL‐P groups. TTN, the largest known human protein, also known as Connectin, acts as a core component of the muscle sarcomere's third filament, being crucial for muscle integrity, function, and elasticity (GeneCards‐TTN 2025). Research has shown that T2DM patients have a decrease in the phosphorylation of specific amino acids in the N2B and PEVK regions of TTN, which leads to increased TTN‐based stiffness (Stroik et al. 2024). This stiffness can be reversed after altered phosphorylation in these domains by improving insulin sensitivity (using metformin or targeting neuregulin‐1). This suggests that TTN stiffness could be a potential therapeutic target for addressing diastolic dysfunction (Stroik et al. 2024).

From the most abundant plasmatic proteins in the (G3)DL‐P group are Gelsolin, CO4A and Vitamin D‐binding protein (Table S3) which are enriched as “Positive regulation of protein processing in phagocytic vesicles” (Figure 5A). However, the mechanisms underlying these proteins in the cited enriched pathway with dyslipidemia and/or periodontitis were not found. Moreover, this is the first study to demonstrate the Nesprin‐1 protein (encoded by the SYNE‐1 gene) in the (G3)DL‐P group, at exclusive and at high levels (log_2_FC = 4.0) in the plasmatic proteome. This protein is expressed in skeletal and smooth muscle, and peripheral blood lymphocyte, and forms a linking network between organelles and the actin cytoskeleton to maintain the subcellular spatial organization. Unfortunately, we could note that there are scarce studies functionally investigating these molecules (e.g., Gelsolin and Nesprin‐1 in the context of dyslipidemia, periodontitis and/or inflammation).

**FIGURE 5:**
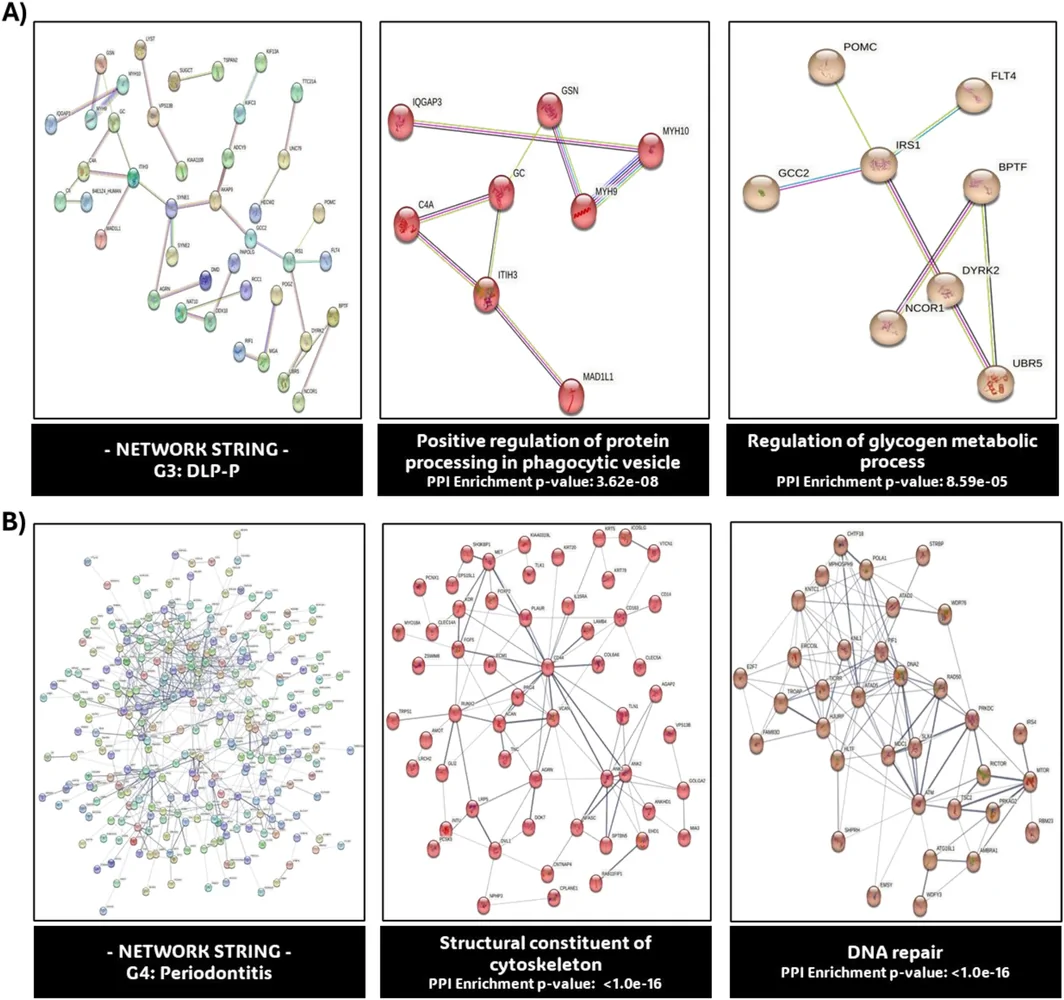
STRING‐db‐based visualization of the protein–protein interaction (PPI) network clustered using the Markov Cluster Algorithm (MCL). (A) Protein–Protein interaction enrichment analysis of up‐regulated (G3)‐DL‐P. (B) Protein–Protein interaction enrichment analysis of up‐regulated (G2)‐Periodontitis. PPI network clustered using the MCL clustering. Inflation parameter: 1,5. For a more detailed view of each protein individually, including protein names, please refer to the Figure S10–S15.

The Ceruloplasmin protein exclusively found in individuals affected by only Perio, is an enzyme that acts in the iron transport across the plasma membrane. It also possesses nitric oxide synthase (NO synthase) and NO‐oxidase (Hochstrasser et al. 2005) activity, being responsible for endogenous nitric oxide (NO) homeostasis (Shiva et al. 2006). Furthermore, it exhibits enzymatic activity similar to that of glutathione peroxidase, which is responsible for detoxifying lipid hydroperoxides and hydrogen peroxide (Park et al. 1999). According to a review by Cardenas et al. (2022), patients with periodontitis showed an increased expression of the ceruloplasmin gene in polymorphonuclear cells when compared to healthy individuals. In agreement, Kido et al. (2012) identified increased levels of this same protein in gingival crevicular fluid samples from periodontal pockets. These findings were corroborated by Harshavardhana et al. (2013), who assessed serum levels of ceruloplasmin and also found them to be elevated in individuals with chronic and aggressive periodontitis. Moreover, the significantly abundant Tenascin‐C protein found in the plasma of (G4)Perio subjects, was also identified at higher levels in human periodontal ligament cells (hPDLCs) under higher tensile or compressive stress compared with those under lower tensile or compressive stress (Xu et al. 2015).

The chronic inflammatory process, caused by T2DM, DL, and Perio, is responsible for promoting pathophysiological oxidative stress by the production of reactive oxygen species (ROS) (Suri et al. 2024), which in turn interact directly with biomolecules, modifying proteins and damaging DNA (Huerta Guevara et al. 2021). Moreover, it was shown that several metabolic pathways were enriched for DNA repair. The chronic hyperglycemic state is believed to be responsible for the generation of free radicals through several pathways and mechanisms, such as the increased activity of the polyol and hexosamine pathways, non‐enzymatic glycation of proteins, activation of protein kinase C, and glucose auto‐oxidation (Rosen et al. 2001; Sliwinska et al. 2008). As reviewed by Rosen et al. (2001), growing evidence demonstrates that the overproduction of ROS is a direct consequence of the hyperglycemic state. This elevates the levels of oxidative stress markers, such as 8‐epi‐prostaglandin F2α (8‐epi‐PGF2α), oxidized lipoprotein (oxLDL), hydroperoxides (ROOH), and 8‐hydroxydeoxyguanosine (8‐OHdG) in individuals with T2DM and T1DM when compared to normoglycemic individuals of the same age (Dandona et al. 1996; Rosen et al. 2001).

Unfortunately, one of the main limitations of our study is that we used a pooled sample for each group and were unable to develop alternative methods (Western Blot, ELISA, or Multiplex), a consequence of the lack of plasma aliquots for such validation. While pooling samples precludes the assessment of inter‐individual biological variation, this approach was utilized to provide a representative overview of the group‐level proteomic profile. It is noteworthy that previous studies, such as the one by Ventura et al. (2018), have demonstrated high consistency between pooled and individual salivary proteome analyses, reporting highly similar results. This suggests that in well‐characterized cohorts, the pooled proteome can reliably reflect the core molecular characteristics of the study group. Other limitations of this study include: (i) the cross‐sectional design; (ii) the absence of control groups for each condition without periodontitis (e.g., T2DM or dyslipidemia alone); (iii) the diagnostic criteria for periodontitis, which limits the validity of the results to the stages III and IV of the disease; (iv) the lack of age‐matching among groups; and (v) the presence of confounding factors inherent to the clinical profiles, such as medication use and obesity. However, these limitations did not compromise the central objective, which was successfully achieved: the presentation of a global and high‐confident plasmatic proteomic analysis of different combinations of T2DM, DL, and Perio, resulting in original findings. This study provides the critical initial data needed to encourage further research and validation of these findings in a larger, more diverse group of individuals. Faithful replication of these findings will crucially depend on strict adherence to the clinical criteria and analytical methods established herein.

## 5 Conclusion

In conclusion, the global plasmatic proteomic analysis demonstrated a significantly altered protein profile in each of the investigated pathological conditions. This specificity provides a foundation for future studies to functionally characterize the identified upregulated proteins, both unique and shared. Further studies are necessary to replicate these findings in larger and more diverse populations, which might contribute to the identification of potential diagnostic and therapeutic targets for each of these disease scenarios.

## Author Contributions

**François Isnaldo Dias Caldeira:** formal analysis, visualization, writing – original draft, writing – review and editing. **Sâmia Cruz Tfaile Corbi:** conceptualization, data curation, methodology, formal analysis, visualization. **Alliny de Souza Bastos:** conceptualization, methodology, visualization, writing – review and editing. **Silvana Regina Perez Orrico:** conceptualization, data curation, visualization, writing – review and editing, methodology. **Cristiane Ribeiro Salmon:** formal analysis, writing – review and editing, visualization. **Yizhi Xiao:** formal analysis, visualization, writing – review and editing. **Walter Luiz Siqueira:** formal analysis, visualization, writing – review and editing, conceptualization, data curation, methodology, funding acquisition. **Raquel Mantuaneli Scarel‐Caminaga:** conceptualization, data curation, formal analysis, visualization, writing – review and editing, methodology, investigation, supervision, funding acquisition.

## Funding

This study was supported by the Foundation for Research Support of State of São Paulo—FAPESP, Grants 2007/08362‐8, 2009/16233‐9, 2014/16148‐0, 2015/08678‐1 and 24/15353‐0. This study was funded by the Saskatchewan Health Research Foundation (SHRF, Grant#5273), Canadian Institutes of Health Research (CIHR; Grant#PJT‐159760, Grant#MOP‐106657), Natural Sciences and Engineering Research Council (NSERC; Grant# 2020/06119) and Canada Foundation for Innovation (Grant#37442).

## Conflicts of Interest

The authors declare no conflicts of interest.

## Supporting information


**Figure S1:** Examples of base‐peak chromatograms of the five groups. Peptide separation was achieved using a nano‐flow reverse‐phase HPLC column, with gradient elution ranging from 5% to 55% solvent B in 85 min. G1: Group 1; G2: Group 2; G3: Group 3; G4: Group 4; G5: Group 5.
**Figure S2:** SDS‐PAGE gel (12%) stained with Coomassie‐blue dye to demonstrate, before and after use of the Depletion Kit, the efficiency of the method. The left column 1 shows the standard molecular weights of proteins (Bio‐Rad Precision Plus All Blue), while the other columns (columns 2, 3, 4, 5 and 6) show the bands formed by the separation of plasma proteins according to their molecular weight and affording to the total protein amount (10–20 μg), and the depleted state (original, depleted plasma sample and Albumin/IgG removed proteins).
**Figure S3:** Enrichment analysis (3A‐Molecular Function) and (3B‐Protein Class) of up‐regulated differentially abundant proteins in each comparison of a diseased group with the Control.
**Figure S4:** Networking String‐G1: T2DMpoorly‐DL‐P. Figure 4A from the main text at higher magnification.
**Figure S5:** Networking String‐G1: T2DMpoorly‐DL‐P. Figure 4A from the main text at higher magnification.
**Figure S6:** Networking String‐G1: T2DMpoorly‐DL‐P. Figure 4A from the main text at higher magnification.
**Figure S7:** Networking String‐G2: T2DMwell‐DL‐P. Figure 4B from the main text at higher magnification.
**Figure S8:** Networking String‐ G2: T2DMwell‐DL‐P. Figure 4B from the main text at higher magnification.
**Figure S9:** Networking String‐ G2: T2DMwell‐DL‐P. Figure 4B from the main text at higher magnification.
**Figure 10**. Networking String‐ G3: DL‐P. Figure 5A from the main text at higher magnification.
**Figure S11:** Networking String‐ G3: DL‐P. Figure 5A from the main text at higher magnification.
**Figure S12:** Networking String‐ G3: DL‐P. Figure 5A from the main text at higher magnification.
**Figure S13:** Networking String‐ G4: Periodontitis Figure 5B from the main text at higher magnification.
**Figure S14:** Networking String‐ G4: Periodontitis Figure 5B from the main text at higher magnification.
**Figure S15:** Networking String‐ G4: Periodontitis Figure 5B from the main text at higher magnification.

**Table S1:** Abundance variation in G1(T2DMpoorly‐DL‐P) versus G5(Control). 25 up‐regulated and 25 down‐regulated proteins.
**Table S2:** Abundance variation in G2(T2DMwell‐DL‐P) versus G5(Control). 25 up‐regulated and 25 down‐regulated proteins.
**Table S3:** Abundance variation in G3(DL‐P) versus G5(Control). 25 up‐regulated and 25 down‐regulated proteins.
**Table S4:** Abundance variation in G4(Perio) versus G5(Control). 25 up‐regulated and 25 down‐regulated proteins.
**Table S5:** Plasmatic proteins exclusive for each group. Quantitative protein values are PSM (spectrum counts).
**Table S6:** Plasmatic proteins in common in all five groups. Quantitative protein values are PSM (spectrum counts).

## Data Availability

The data that support the findings of this study are available on request from the corresponding author. The data are not publicly available due to privacy or ethical restrictions.

## References

[odi70313-bib-0001] Afzal, S. , S. E. Bojesen , and B. G. Nordestgaard . 2013. “Low 25‐Hydroxyvitamin D and Risk of Type 2 Diabetes: A Prospective Cohort Study and Meta‐Analysis.” Clinical Chemistry 59, no. 2: 381–391. 10.1373/clinchem.2012.193003. 23232064

[odi70313-bib-0002] Agho, E. T. , F. J. Owotade , B. A. Kolawole , E. O. Oyetola , and T. A. Adedeji . 2021. “Salivary Inflammatory Biomarkers and Glycated Haemoglobin Among Patients With Type 2 Diabetic Mellitus.” BMC Oral Health 21, no. 1: 101. 10.1186/s12903-021-01453-y. PMC8095344 33676486

[odi70313-bib-0003] Athyros, V. G. , M. Doumas , K. P. Imprialos , et al. 2018. “Diabetes and Lipid Metabolism.” Hormones (Athens, Greece) 17, no. 1: 61–67. 10.1007/s42000-018-0014-8. 29858856

[odi70313-bib-0004] Brohee, S. , and J. van Helden . 2006. “Evaluation of Clustering Algorithms for Protein‐Protein Interaction Networks.” BMC Bioinformatics 7: 488. 10.1186/1471-2105-7-488. PMC1637120 17087821

[odi70313-bib-0005] Buchwald, S. , T. Kocher , R. Biffar , A. Harb , B. Holtfreter , and P. Meisel . 2013. “Tooth Loss and Periodontitis by Socio‐Economic Status and Inflammation in a Longitudinal Population‐Based Study.” Journal of Clinical Periodontology 40, no. 3: 203–211. 10.1111/jcpe.12056. 23379538

[odi70313-bib-0006] Cai, X. , Z. Xue , F. F. Zeng , et al. 2023. “Population Serum Proteomics Uncovers a Prognostic Protein Classifier for Metabolic Syndrome.” Cell Reports Medicine 4, no. 9: 101172. 10.1016/j.xcrm.2023.101172. PMC10518601 37652016

[odi70313-bib-0007] Cardenas, A. M. , L. J. Ardila , R. Vernal , S. Melgar‐Rodriguez , and H. G. Hernandez . 2022. “Biomarkers of Periodontitis and Its Differential DNA Methylation and Gene Expression in Immune Cells: A Systematic Review.” International Journal of Molecular Sciences 23, no. 19: 1–18. 10.3390/ijms231912042. PMC9570497 36233348

[odi70313-bib-0008] Carvalho, J. G. , A. D. Leite , C. Peres‐Buzalaf , et al. 2013. “Renal Proteome in Mice With Different Susceptibilities to Fluorosis.” PLoS One 8, no. 1: e53261. 10.1371/journal.pone.0053261. PMC3537663 23308176

[odi70313-bib-0009] Cheng, J. , H. P. Liu , S. L. Hwang , L. F. Hsu , W. Y. Lin , and F. J. Tsai . 2019. “Dystonin/BPAG1 Modulates Diabetes and Alzheimer's Disease Cross‐Talk: A Meta‐Analysis.” Neurological Sciences 40, no. 8: 1577–1582. 10.1007/s10072-019-03879-3. 30963337

[odi70313-bib-0010] Corbi, S. C. T. , J. F. de Vasconcellos , A. S. Bastos , et al. 2020. “Circulating Lymphocytes and Monocytes Transcriptomic Analysis of Patients With Type 2 Diabetes Mellitus, Dyslipidemia and Periodontitis.” Scientific Reports 10, no. 1: 8145. 10.1038/s41598-020-65042-9. PMC7235087 32424199

[odi70313-bib-0011] Cuschieri, S. 2019. “The STROBE Guidelines.” Saudi Journal of Anaesthesia 13, no. Suppl 1: S31–S34. 10.4103/sja.SJA_543_18. PMC6398292 30930717

[odi70313-bib-0012] Dandona, P. , K. Thusu , S. Cook , et al. 1996. “Oxidative Damage to DNA in Diabetes Mellitus.” Lancet 347, no. 8999: 444–445. 10.1016/s0140-6736(96)90013-6. 8618487

[odi70313-bib-0013] Deutsch, E. W. , G. S. Omenn , Z. Sun , et al. 2021. “Advances and Utility of the Human Plasma Proteome.” Journal of Proteome Research 20, no. 12: 5241–5263. 10.1021/acs.jproteome.1c00657. PMC9469506 34672606

[odi70313-bib-0014] Emilsson, V. , M. Ilkov , J. R. Lamb , et al. 2018. “Co‐Regulatory Networks of Human Serum Proteins Link Genetics to Disease.” Science 361, no. 6404: 769–773. 10.1126/science.aaq1327. PMC6190714 30072576

[odi70313-bib-0015] Feng, X. , Q. Guo , S. Zhou , et al. 2020. “Could Remnant‐Like Particle Cholesterol Become a Risk Factor in Diabetic Menopausal Women With Coronary Artery Disease? A Cross‐Sectional Study of a Single Academic Center in China.” Lipids in Health and Disease 19, no. 1: 44. 10.1186/s12944-020-01224-8. PMC7076918 32178671

[odi70313-bib-0016] Frick, E. A. , V. Emilsson , T. Jonmundsson , et al. 2024. “Serum Proteomics Reveal APOE‐ε4‐Dependent and APOE‐ε4‐Independent Protein Signatures in Alzheimer's Disease.” Nature Aging 4, no. 10: 1446–1464. 10.1038/s43587-024-00693-1. PMC11485263 39169269

[odi70313-bib-0017] Fujita, T. , S. Hemmi , M. Kajiwara , et al. 2013. “Complement‐Mediated Chronic Inflammation Is Associated With Diabetic Microvascular Complication.” Diabetes/Metabolism Research and Reviews 29, no. 3: 220–226. 10.1002/dmrr.2380. 23280928

[odi70313-bib-0018] GeneCards . 2025. “Vitamin D‐Binding Protein.” GeneCards: The Human Gene Database. City of Hope/Weizmann Institute of Science. https://www.genecards.org/cgi‐bin/carddisp.pl?gene=GC.

[odi70313-bib-0019] Gonzalez‐Lleo, A. M. , R. M. Sanchez‐Hernandez , M. Boronat , and A. M. Wagner . 2022. “Diabetes and Familial Hypercholesterolemia: Interplay Between Lipid and Glucose Metabolism.” Nutrients 14, no. 7: 1–23. 10.3390/nu14071503. PMC9002616 35406116

[odi70313-bib-0020] Gross, B. , M. Pawlak , P. Lefebvre , and B. Staels . 2017. “PPARs in Obesity‐Induced T2DM, Dyslipidaemia and NAFLD.” Nature Reviews Endocrinology 13, no. 1: 36–49. 10.1038/nrendo.2016.135. 27636730

[odi70313-bib-0021] Harshavardhana, B. , S. K. Rath , and M. Mukherjee . 2013. “Evaluation of Serum Ceruloplasmin in Aggressive and Chronic Periodontitis Patients.” Journal of Indian Society of Periodontology 17, no. 3: 333–337. 10.4103/0972-124X.115659. PMC3768184 24049334

[odi70313-bib-0022] Heitz‐Mayfield, L. J. 2005. “Disease Progression: Identification of High‐Risk Groups and Individuals for Periodontitis.” Journal of Clinical Periodontology 32, no. 6: 196–209. 10.1111/j.1600-051X.2005.00803.x. 16128838

[odi70313-bib-0023] Hochstrasser, H. , J. Tomiuk , U. Walter , et al. 2005. “Functional Relevance of Ceruloplasmin Mutations in Parkinson's Disease.” FASEB Journal 19, no. 13: 1851–1853. 10.1096/fj.04-3486fje. 16150804

[odi70313-bib-0024] Huerta Guevara, A. P. , S. J. McGowan , M. Kazantzis , et al. 2021. “Increased Insulin Sensitivity and Diminished Pancreatic Beta‐Cell Function in DNA Repair Deficient Ercc1(d/−) Mice.” Metabolism 117: 154711. 10.1016/j.metabol.2021.154711. PMC8625516 33493548

[odi70313-bib-0025] Joshi, P. H. , A. A. Khokhar , J. M. Massaro , et al. 2016. “Remnant Lipoprotein Cholesterol and Incident Coronary Heart Disease: The Jackson Heart and Framingham Offspring Cohort Studies.” Journal of the American Heart Association 5, no. 5: 1–21. 10.1161/JAHA.115.002765. PMC4889167 27130348

[odi70313-bib-0026] Kido, J. , M. Bando , Y. Hiroshima , et al. 2012. “Analysis of Proteins in Human Gingival Crevicular Fluid by Mass Spectrometry.” Journal of Periodontal Research 47, no. 4: 488–499. 10.1111/j.1600-0765.2011.01458.x. 22220998

[odi70313-bib-0027] Li, C. , J. Xiao , S. Wu , et al. 2023. “Clinical Application of Serum‐Based Proteomics Technology in Human Tumor Research.” Analytical Biochemistry 663: 115031. 10.1016/j.ab.2022.115031. 36580994

[odi70313-bib-0028] Li, Y. , B. Wang , F. Ma , et al. 2025. “Data‐Independent Acquisition‐Based Quantitative Proteomics for Pairwise Comparison of Serum and Plasma.” Journal of Proteome Research 24, no. 6: 2614–2627. 10.1021/acs.jproteome.4c00783. 40402807

[odi70313-bib-0029] Loe, H. 1993. “Periodontal Disease: The Sixth Complication of Diabetes Mellitus.” Diabetes Care 16, no. 1: 329–334. https://www.ncbi.nlm.nih.gov/pubmed/8422804. 8422804

[odi70313-bib-0030] Mealey, B. L. , T. W. Oates , and American Academy of Periodontology . 2006. “Diabetes Mellitus and Periodontal Diseases.” Journal of Periodontology 77, no. 8: 1289–1303. 10.1902/jop.2006.050459. 16881798

[odi70313-bib-0031] Muscari, A. , C. Bozzoli , G. M. Puddu , et al. 1995. “Association of Serum C3 Levels With the Risk of Myocardial Infarction.” American Journal of Medicine 98, no. 4: 357–364. 10.1016/S0002-9343(99)80314-3. 7709948

[odi70313-bib-0032] National Cholesterol Education Program Expert Panel on Detection, Evaluation, and Treatment of High Blood Cholesterol in Adults . 2002. “Third Report of the National Cholesterol Education Program (NCEP) Expert Panel on Detection, Evaluation, and Treatment of High Blood Cholesterol in Adults (Adult Treatment Panel III) Final Report.” Circulation 106, no. 25: 3143–3421. https://www.ncbi.nlm.nih.gov/pubmed/12485966. 12485966

[odi70313-bib-0033] Nepomuceno, R. , B. F. Vallerini , R. L. da Silva , et al. 2019. “Systemic Expression of Genes Related to Inflammation and Lipid Metabolism in Patients With Dyslipidemia, Type 2 Diabetes Mellitus and Chronic Periodontitis.” Diabetes and Metabolic Syndrome: Clinical Research and Reviews 13, no. 4: 2715–2722. 10.1016/j.dsx.2019.07.003. 31405698

[odi70313-bib-0034] Oliveros, J. C. 2015. “VENNY: An Interactive Tool for Comparing Lists With Venn Diagrams.”

[odi70313-bib-0035] Park, Y. S. , K. Suzuki , N. Taniguchi , and J. M. Gutteridge . 1999. “Glutathione Peroxidase‐Like Activity of Caeruloplasmin as an Important Lung Antioxidant.” FEBS Letters 458, no. 2: 133–136. 10.1016/s0014-5793(99)01142-4. 10481051

[odi70313-bib-0036] Reckelkamm, S. L. , I. Kaminska , S. E. Baumeister , et al. 2023. “Targeted Proteomics in a Population‐Based Study Identifies Serum PECAM‐1 and TRIM21 as Inflammation Markers for Periodontitis.” Clinical Oral Investigations 28, no. 1: 59. 10.1007/s00784-023-05442-z. PMC10756891 38157091

[odi70313-bib-0037] Rosen, P. , P. P. Nawroth , G. King , W. Moller , H. J. Tritschler , and L. Packer . 2001. “The Role of Oxidative Stress in the Onset and Progression of Diabetes and Its Complications: A Summary of a Congress Series Sponsored by UNESCO‐MCBN, the American Diabetes Association and the German Diabetes Society.” Diabetes/Metabolism Research and Reviews 17, no. 3: 189–212. 10.1002/dmrr.196. 11424232

[odi70313-bib-0038] Saeedi, P. , I. Petersohn , P. Salpea , et al. 2019. “Global and Regional Diabetes Prevalence Estimates for 2019 and Projections for 2030 and 2045: Results From the International Diabetes Federation Diabetes Atlas, 9th Edition.” Diabetes Research and Clinical Practice 157: 107843. 10.1016/j.diabres.2019.107843. 31518657

[odi70313-bib-0039] Sarkar, S. , E. C. Elliott , H. R. Henry , et al. 2023. “Systematic Review of Type 1 Diabetes Biomarkers Reveals Regulation in Circulating Proteins Related to Complement, Lipid Metabolism, and Immune Response.” Clinical Proteomics 20, no. 1: 38. 10.1186/s12014-023-09429-6. PMC10512508 37735622

[odi70313-bib-0040] Shannon, P. , A. Markiel , O. Ozier , et al. 2003. “Cytoscape: A Software Environment for Integrated Models of Biomolecular Interaction Networks.” Genome Research 13, no. 11: 2498–2504. 10.1101/gr.1239303. PMC403769 14597658

[odi70313-bib-0041] Shiva, S. , X. Wang , L. A. Ringwood , et al. 2006. “Ceruloplasmin Is a NO Oxidase and Nitrite Synthase That Determines Endocrine NO Homeostasis.” Nature Chemical Biology 2, no. 9: 486–493. 10.1038/nchembio813. 16906150

[odi70313-bib-0042] Siqueira, W. L. , and C. Dawes . 2011. “The Salivary Proteome: Challenges and Perspectives.” Proteomics: Clinical Applications 5, no. 11–12: 575–579. 10.1002/prca.201100046. 21956964

[odi70313-bib-0043] Sliwinska, A. , J. Blasiak , J. Kasznicki , and J. Drzewoski . 2008. “In Vitro Effect of Gliclazide on DNA Damage and Repair in Patients With Type 2 Diabetes Mellitus (T2DM).” Chemico‐Biological Interactions 173, no. 3: 159–165. 10.1016/j.cbi.2008.03.017. 18485337

[odi70313-bib-0044] Stanley, B. A. , R. L. Gundry , R. J. Cotter , and J. E. Van Eyk . 2004. “Heart Disease, Clinical Proteomics and Mass Spectrometry.” Disease Markers 20, no. 3: 167–178. 10.1155/2004/965261. PMC3839266 15502250

[odi70313-bib-0045] Stroik, D. , Z. R. Gregorich , F. Raza , Y. Ge , and W. Guo . 2024. “Titin: Roles in Cardiac Function and Diseases.” Frontiers in Physiology 15: 1385821. 10.3389/fphys.2024.1385821. PMC11040099 38660537

[odi70313-bib-0046] Suh, S. , and K. W. Kim . 2011. “Diabetes and Cancer: Is Diabetes Causally Related to Cancer?” Diabetes & Metabolism Journal 35, no. 3: 193–198. 10.4093/dmj.2011.35.3.193. PMC3138100 21785737

[odi70313-bib-0047] Suri, S. , P. Mitra , A. Abhilasha , et al. 2024. “Altered Expression of Specific Antioxidant (SOD1 and SOD2) and DNA Repair (XRCC1 and OGG1) Genes in Patients With Newly Diagnosed Type‐2 Diabetes Mellitus.” Minerva Endocrinology 49, no. 4: 398–405. 10.23736/S2724-6507.21.03417-5. 34160187

[odi70313-bib-0048] Szklarczyk, D. , R. Kirsch , M. Koutrouli , et al. 2023. “The STRING Database in 2023: Protein‐Protein Association Networks and Functional Enrichment Analyses for Any Sequenced Genome of Interest.” Nucleic Acids Research 51, no. D1: D638–D646. 10.1093/nar/gkac1000. PMC9825434 36370105

[odi70313-bib-0049] Taylor, G. W. 2001. “Bidirectional Interrelationships Between Diabetes and Periodontal Diseases: An Epidemiologic Perspective.” Annals of Periodontology 6, no. 1: 99–112. 10.1902/annals.2001.6.1.99. 11887478

[odi70313-bib-0050] Tilton, R. G. , S. J. Haidacher , W. S. Lejeune , et al. 2007. “Diabetes‐Induced Changes in the Renal Cortical Proteome Assessed With Two‐Dimensional Gel Electrophoresis and Mass Spectrometry.” Proteomics 7, no. 10: 1729–1742. 10.1002/pmic.200700017. 17436268

[odi70313-bib-0051] Tonetti, M. S. , H. Greenwell , and K. S. Kornman . 2018. “Staging and Grading of Periodontitis: Framework and Proposal of a New Classification and Case Definition.” Journal of Periodontology 89, no. 1: S159–S172. 10.1002/JPER.18-0006. 29926952

[odi70313-bib-0052] Turkes, G. F. , S. Uysal , T. Demir , et al. 2021. “Associations Between Bioavailable Vitamin D and Remnant Cholesterol in Patients With Type 2 Diabetes Mellitus.” Cureus 13, no. 2: e13248. 10.7759/cureus.13248. PMC7948319 33728195

[odi70313-bib-0053] Ventura, T. , N. R. Ribeiro , A. S. Dionizio , I. T. Sabino , and M. A. R. Buzalaf . 2018. “Standardization of a Protocol for Shotgun Proteomic Analysis of Saliva.” Journal of Applied Oral Science 26: e20170561. 10.1590/1678-7757-2017-0561. PMC6007968 29898185

[odi70313-bib-0054] Wang, W. , Z. Shi , R. Zhang , et al. 2021. “Liver Proteomics Analysis Reveals Abnormal Metabolism of Bile Acid and Arachidonic Acid in Chinese Hamsters With Type 2 Diabetes Mellitus.” Journal of Proteomics 239: 104186. 10.1016/j.jprot.2021.104186. 33722748

[odi70313-bib-0055] Watt, R. G. , J. G. Steele , E. T. Treasure , D. A. White , N. B. Pitts , and J. J. Murray . 2013. “Adult Dental Health Survey 2009: Implications of Findings for Clinical Practice and Oral Health Policy.” British Dental Journal 214, no. 2: 71–75. 10.1038/sj.bdj.2013.50. 23348457

[odi70313-bib-0056] World Health Organization . 2000. “Obesity: Preventing and Managing the Global Epidemic. Report of a WHO Consultation.” World Health Organization Technical Report Series 894: 1–253. https://www.ncbi.nlm.nih.gov/pubmed/11234459. 11234459

[odi70313-bib-0057] Xu, H. , D. Bai , L. B. Ruest , et al. 2015. “Expression Analysis of Alpha‐Smooth Muscle Actin and Tenascin‐C in the Periodontal Ligament Under Orthodontic Loading or In Vitro Culture.” International Journal of Oral Science 7, no. 4: 232–241. 10.1038/ijos.2015.26. PMC5153592 26674425

[odi70313-bib-0058] Yin, L. , G. Feng , C. Huang , and W. Cai . 2022. “Proteomic Analysis of Serum Lysine Acetylation in Uyghur Patients With T2DM.” Frontiers in Molecular Biosciences 9: 787885. 10.3389/fmolb.2022.787885. PMC9006524 35433838

[odi70313-bib-0059] Zhao, Y. X. , S. Borjigin , and Z. L. Yan . 2022. “Functional Annotation and Enrichment Analysis of Differentially Expressed Serum Proteins in Patients With Type 2 Diabetes After Dapagliflozin.” World Journal of Diabetes 13, no. 3: 224–239. 10.4239/wjd.v13.i3.224. PMC8984562 35432754

[odi70313-bib-0060] Zhou, Y. , B. Zhou , L. Pache , et al. 2019. “Metascape Provides a Biologist‐Oriented Resource for the Analysis of Systems‐Level Datasets.” Nature Communications 10, no. 1: 1523. 10.1038/s41467-019-09234-6. PMC6447622 30944313

