# Supplementary material for: Plasmatic Profiling of Individuals With Combinations of Type 2 Diabetes Mellitus, Dyslipidemia and Periodontitis: A Cross‐Sectional Study
Source: Oral Dis. 2026 Apr 3;32(8):2465–79. doi: 10.1111/odi.70313 (PMC13693639; doi:10.1111/odi.70313)
Supplement: Supplementary file 1 [file ODI-32-2465-s001.docx]

**Supplementary material – Plasma proteome profiling**

**Results of In-solution digestion. Protein identification**

In this first proteomics approach, aliquot (pool plasma samples of G1, G2, G3, G4 and G5 - 20 μg of protein each) were subjected to in-solution digestion with trypsin, desalting with ZipTip C18 and analyzed with MS (a total of 5 runs per group were performed). The base-peak chromatogram for reversed-phase chromatography monitored by the mass spectrometer represents the intensity of all peptide’s ions in the sample in a single scan. Plasma proteome from all five groups showed a consistent elution of protein/peptides range from 10 to 60 min (Supplementary Figure 1).

**Supplementary Figure 1.** Examples of base-peak chromatograms of the five groups. Peptide separation was achieved using a nano-flow reverse-phase HPLC column, with gradient elution ranging from 5 to 55% solvent B in 85 min. G1: Group 1; G2: Group 2; G3: Group 3; G4: Group 4; G5: Group 5.

Supplementary Figure 2 showed the SDS-PAGE gel test (12%), before and after the use of Albumin and IgG Depletion Kit (with the original, depleted plasma sample and Albumin/IgG removed proteins). Also, figure 6 shows that the sample containing of 20 ug of total proteins concentration presented the best result in the SDS-PAGE gel test (12%) after the electrophoresis and stained with Coomassie-blue dye.

**Supplementary Figure 2.** SDS-PAGE gel (12%) stained with Coomassie-blue dye to demonstrate, before and after use of the Depletion Kit, the efficiency of the method. The left column 1 shows the standard molecular weights of proteins (Bio-Rad Precision Plus All Blue), while the other columns (columns 2, 3, 4, 5 and 6) show the bands formed by the separation of plasma proteins according to their molecular weight and affording to the total protein amount (10 – 20 ug), and the depleted state (original, depleted plasma sample and Albumin/IgG removed proteins).

After removing the large amount of these two plasma proteins (Albumin and IgG), the measurement of total protein concentration of each pool plasma sample (from each five groups) was performed by the bicinchoninic acid assay (BCA - Thermo Pierce, Rockford, IL, USA). Total protein concentrations of pool plasma samples (BCA – µg/ml) did not present statistical difference amongst groups (T-test, α=5%): (G1)=1643.25 µg/ml, (G2)= 1736.27 µg/ml, (G3)= 1567.63 µg/ml, (G4)= 1541.16 µg/ml, (G5)= 1594.0 µg/ml.

 GO enrichment analyses were performed in PANTHER GO-Slim for the additional functional interpretation of the up regulated proteins with differential abundance in each diseased group compared to the control group, according to Molecular Function and Protein Class terms (Supplementary Figure 3). (G4)Perio presented the highest number of enriched proteins for the MF binding term, being “binding” the most enriched GO MF term with 73 proteins, followed by the terms “Catalytic activity” and “Transcription regulator activity” with 30 and 14 proteins, respectively (Supplementary Figure 3A). Regarding GO PC terms, the most enriched was the (G4)Perio, with 15 proteins for “cytoskeletal protein”, 14 proteins for “scaffold adaptor protein” and 12 proteins for “Transporter” (Supplementary Figure 3B).

**B)**

**A)**

**Supplementary Figure 3.** Enrichment analysis (3A- Molecular Function) and (3B-Protein Class) of up-regulated differentially abundant proteins in each comparison of a diseased group with the Control.

**Supplementary Figure 4.** Networking String- G1: T2DMpoorly-DL-P. Figure 4A from the main text at higher magnification

Protein-Protein interaction enrichment analysis of up-regulated (G1)-T2DMpoorly-DL-P. PPI network clustered using the MCL clustering. Inflation parameter: 1,5.

**Supplementary Figure 5.** Networking String- G1: T2DMpoorly-DL-P. Figure 4A from the main text at higher magnification

Protein-Protein interaction enrichment analysis of up-regulated (G1)-T2DMpoorly-DL-P. PPI network clustered using the MCL clustering. Inflation parameter: 1,5.

**Supplementary Figure 6.** Networking String- G1: T2DMpoorly-DL-P. Figure 4A from the main text at higher magnification

Protein-Protein interaction enrichment analysis of up-regulated (G1)-T2DMpoorly-DL-P. PPI network clustered using the MCL clustering. Inflation parameter: 1,5.

**Supplementary Figure 7.** Networking String- G2: T2DMwell-DL-P. Figure 4B from the main text at higher magnification

Protein-Protein interaction enrichment analysis of up-regulated (G2)-T2DMwell-DL-P. PPI network clustered using the MCL clustering. Inflation parameter: 1,5.

**Supplementary Figure 8.** Networking String- G2: T2DMwell-DL-P. Figure 4B from the main text at higher magnification

Protein-Protein interaction enrichment analysis of up-regulated (G2)-T2DMwell-DL-P. PPI network clustered using the MCL clustering. Inflation parameter: 1,5.

**Supplementary Figure 9.** Networking String- G2: T2DMwell-DL-P. Figure 4B from the main text at higher magnification

Protein-Protein interaction enrichment analysis of up-regulated (G2)-T2DMwell-DL-P. PPI network clustered using the MCL clustering. Inflation parameter: 1,5.

**Supplementary Figure 10.** Networking String- G3: DL-P. Figure 5A from the main text at higher magnification

Protein-Protein interaction enrichment analysis of up-regulated (G3)-DL-P. PPI network clustered using the MCL clustering. Inflation parameter: 1,5

**Supplementary Figure 11.** Networking String- G3: DL-P. Figure 5A from the main text at higher magnification

Protein-Protein interaction enrichment analysis of up-regulated (G3)-DL-P. PPI network clustered using the MCL clustering. Inflation parameter: 1,5

**Supplementary Figure 12.** Networking String- G3: DL-P. Figure 5A from the main text at higher magnification

Protein-Protein interaction enrichment analysis of up-regulated (G3)-DL-P. PPI network clustered using the MCL clustering. Inflation parameter: 1,5

**Supplementary Figure 13.** Networking String- G4: Periodontitis Figure 5B from the main text at higher magnification

Protein-Protein interaction enrichment analysis of up-regulated G2)-Periodontitis. PPI network clustered using the MCL clustering. Inflation parameter: 1,5

**Supplementary Figure 14.** Networking String- G4: Periodontitis Figure 5B from the main text at higher magnification

Protein-Protein interaction enrichment analysis of up-regulated G2)-Periodontitis. PPI network clustered using the MCL clustering. Inflation parameter: 1,5

**Supplementary Figure 15.** Networking String- G4: Periodontitis Figure 5B from the main text at higher magnification

Protein-Protein interaction enrichment analysis of up-regulated G2)-Periodontitis. PPI network clustered using the MCL clustering. Inflation parameter: 1,5
