# Supplementary material for: Plasmatic Profiling of Individuals With Combinations of Type 2 Diabetes Mellitus, Dyslipidemia and Periodontitis: A Cross‐Sectional Study
Source: Oral Dis. 2026 Apr 3;32(8):2465–79. doi: 10.1111/odi.70313 (PMC13693639; doi:10.1111/odi.70313)
Supplement: Supplementary file 2 [file ODI-32-2465-s002.docx]

| **Supplementary Table 1.** Abundance variation in G1(T2DMpoorly-DL-P) versus G5(Control). 25 up-regulated and 25 down-regulated proteins. | | | | | | | | | |  |  | |  |
| --- | --- | --- | --- | --- | --- | --- | --- | --- | --- | --- | --- | --- | --- |
| **Entry** | **Entry Name UNIPROT** | | | | **Protein names** | **Gene Names** | **Length** | | **Mass** | **Score** | |  | |
| P0C0L4 | CO4A_HUMAN | | | | Complement C4-A | C4A, CO4, CPAMD2 | 1,744 AA | | 192,785 Da | 6,73 | | ↑ | |
| Q6N092 | Q6N092_HUMAN | | | | Uncharacterized protein DKFZp686K18196 | DKFZp686K18196 | 519 AA | | 56,424 Da | 6,09 | | ↑ | |
| Q8WZ42 | TITIN_HUMAN | | | | Titin | TTN | 34,350 AA | | 3,816,030 Da | 5,70 | | ↑ | |
| Q9NRC6 | SPTN5_HUMAN | | | | Spectrin beta chain, non-erythrocytic 5 | SPTBN5, BSPECV, HUBSPECV, HUSPECV | 3,674 AA | | 416,750 Da | 5,13 | | ↑ | |
| A0A0A0MS51 | A0A0A0MS51_HUMAN | | | | Gelsolin | GSN | 748 AA | | 82,526 Da | 4,81 | | ↑ | |
| A4D1A8 | A4D1A8_HUMAN | | | | Similar to Piccolo protein (Aczonin) | LOC392742, tcag7.1316 | 3,717 AA | | 411,161 Da | 4,75 | | ↑ | |
| B4E1C2 | B4E1C2_HUMAN | | | | Kininogen 1, isoform CRA_b | KNG1, hCG_2021397 | 644 AA | | 71,915 Da | 4,75 | | ↑ | |
| Q9HCM1 | RESF1_HUMAN | | | | Retroelement silencing factor 1 | RESF1, C12orf35, KIAA1551 | 1,747 AA | | 194,857 Da | 4,75 | | ↑ | |
| B7ZLC8 | B7ZLC8_HUMAN | | | | Probable JmjC domain-containing histone demethylation protein 2C | JMJD1C | 2,358 AA | | 263,155 Da | 4,46 | | ↑ | |
| E9PCK9 | E9PCK9_HUMAN | | | | Muscular LMNA interacting protein | MLIP | 561 AA | | 59,112 Da | 4,39 | | ↑ | |
| Q7Z7G8 | VP13B_HUMAN | | | | Intermembrane lipid transfer protein VPS13B | VPS13B, CHS1, COH1, KIAA0532 | 4,022 AA | | 448,664 Da | 4,39 | | ↑ | |
| Q8N7H6 | Q8N7H6_HUMAN | | | | cDNA FLJ25555 fis, clone JTH02535 |  | 217 AA | | 23,041 Da | 4,39 | | ↑ | |
| E7EPM4 | E7EPM4_HUMAN | | | | Mucin 17, cell surface associated | MUC17 | 4,262 AA | | 425,553 Da | 4,39 | | ↑ | |
| Q5T481 | RBM20_HUMAN | | | | RNA-binding protein 20 | RBM20 | 1,227 AA | | 134,258 Da | 4,25 | | ↑ | |
| F8VWH5 | F8VWH5_HUMAN | | | | Sodium channel epithelial 1 subunit delta | SCNN1D | 328 AA | | 35,070 Da | 4,25 | | ↑ | |
| H0YMD1 | H0YMD1_HUMAN | | | | Low-density lipoprotein receptor | LDLR | 948 AA | | 104,755 Da | 4,17 | | ↑ | |
| O75095 | MEGF6_HUMAN | | | | Multiple epidermal growth factor-like domains protein 6 | MEGF6, EGFL3, KIAA0815 | 1,541 AA | | 161,185 Da | 4,17 | | ↑ | |
| G3V3S8 | G3V3S8_HUMAN | | | | RNA binding motif protein 23 | RBM23 | 66 AA | | 7,365 Da | 4,17 | | ↑ | |
| O15265 | ATX7_HUMAN | | | | Ataxin-7 | ATXN7, SCA7 | 892 AA | | 95,451 Da | 4,17 | | ↑ | |
| Q6ZMT4 | KDM7A_HUMAN | | | | Lysine-specific demethylase 7A | KDM7A, JHDM1D, KDM7, KIAA1718 | 941 AA | | 106,557 Da | 4,09 | | ↑ | |
| J3KQ66 | J3KQ66_HUMAN | | | | Reelin | RELN | 3,469 AA | | 388,989 Da | 4,09 | | ↑ | |
| Q9NZJ4 | SACS_HUMAN | | | | Sacsin | SACS, DNAJC29, KIAA0730 | 4,579 AA | | 521,126 Da | 4,09 | | ↑ | |
| Q68DA7 | FMN1_HUMAN | | | | Formin-1 | FMN1, FMN, LD | 1,419 AA | | 157,578 Da | 4,09 | | ↑ | |
| Q5VYK3 | ECM29_HUMAN | | | | Proteasome adapter and scaffold protein ECM29 | ECPAS, ECM29, KIAA0368 | 1,845 AA | | 204,291 Da | 4,00 | | ↑ | |
| Q5T985 | Q5T985_HUMAN | | | | Inter-alpha-trypsin inhibitor heavy chain 2 | ITIH2 | 935 AA | | 105,216 Da | -6,07 | | ↓ | |
| P01876 | IGHA1_HUMAN | | | | Immunoglobulin heavy constant alpha 1 | IGHA1 | 398 AA | | 42,849 Da | -5,95 | | ↓ | |
| P00751 | CFAB_HUMAN | | | | Complement factor B | CFB, BF, BFD | 764 AA | | 85,533 Da | -5,93 | | ↓ | |
| H6VRF8 | H6VRF8_HUMAN | | | | Keratin, type II cytoskeletal 1 | KRT1 | 644 AA | | 66,053 Da | -5,32 | | ↓ | |
| T1R7A8 | T1R7A8_HUMAN | | | | MUC5AC | MUC5AC | 3,483 AA | | 350,921 Da | -5,17 | | ↓ | |
| B7Z992 | B7Z992_HUMAN | | | | Gelsolin |  | 715 AA | | 78,832 Da | -5,13 | | ↓ | |
| B4DPP8 | B4DPP8_HUMAN | | | | cDNA FLJ53075, highly similar to Kininogen-1 |  | 415 AA | | 46,496 Da | -4,95 | | ↓ | |
| A0A0A0MRF6 | A0A0A0MRF6_HUMAN | | | | A-kinase anchoring protein 9 | AKAP9 | 3,931 AA | | 455,919 Da | -4,86 | | ↓ | |
| B4DMR2 | B4DMR2_HUMAN | | | | cDNA FLJ50582, highly similar to Cyclin-dependent kinase-like 3 |  | 185 AA | | 20,689 Da | -4,75 | | ↓ | |
| P13645 | K1C10_HUMAN | | | | Keratin, type I cytoskeletal 10 | KRT10, KPP | 584 AA | | 58,827 Da | -4,64 | | ↓ | |
| J3KNM6 | J3KNM6_HUMAN | | | | Arf-GAP with GTPase, ANK repeat and PH domain-containing protein 2 | AGAP2 | 1,036 AA | | 108,641 Da | -4,64 | | ↓ | |
| G3V0E6 | G3V0E6_HUMAN | | | | Podocalyxin like | PODXL, hCG_18687 | 324 AA | | 32,702 Da | -4,58 | | ↓ | |
| B5MCN7 | B5MCN7_HUMAN | | | | Nuclear receptor coactivator 1 | NCOA1 | 1,248 AA | | 135,621 Da | -4,58 | | ↓ | |
| D6RFH5 | D6RFH5_HUMAN | | | | Folliculin interacting protein 2 | FNIP2 | 673 AA | | 74,715 Da | -4,52 | | ↓ | |
| P35908 | K22E_HUMAN | | | | Keratin, type II cytoskeletal 2 epidermal | KRT2, KRT2A, KRT2E | 639 AA | | 65,433 Da | -4,52 | | ↓ | |
| Q9Y5S2 | MRCKB_HUMAN | | | | Serine/threonine-protein kinase MRCK beta | CDC42BPB, KIAA1124 | 1,711 AA | | 194,315 Da | -4,52 | | ↓ | |
| Q96N23 | CFA54_HUMAN | | | | Cilia- and flagella-associated protein 54 | CFAP54, C12orf55, C12orf63 | 3,096 AA | | 351,970 Da | -4,46 | | ↓ | |
| E9PFQ4 | E9PFQ4_HUMAN | | | | Pleckstrin homology like domain family B member 2 | PHLDB2 | 1,115 AA | | 125,476 Da | -4,46 | | ↓ | |
| H3BRH3 | H3BRH3_HUMAN | | | | Zinc finger CCCH-type containing 18 | ZC3H18 | 196 AA | | 21,009 Da | -4,46 | | ↓ | |
| Q5M775 | CYTSB_HUMAN | | | | Cytospin-B | SPECC1, CYTSB, NSP5 | 1,068 AA | | 118,585 Da | -4,39 | | ↓ | |
| Q5CZC0 | FSIP2_HUMAN | | | | Fibrous sheath-interacting protein 2 | FSIP2 | 6,907 AA | | 780,607 Da | -4,25 | | ↓ | |
| B3KRD6 | B3KRD6_HUMAN | | | | cDNA FLJ34074 fis, clone FCBBF3002925, highly similar to Mus musculus SET domain containing 5 (Setd5), mRNA |  | 1,344 AA | | 147,456 Da | -4,25 | | ↓ | |
| Q14669 | TRIPC_HUMAN | | | | E3 ubiquitin-protein ligase TRIP12 | TRIP12, KIAA0045, ULF | 1,992 AA | | 220,434 Da | -4,25 | | ↓ | |
| Q8IWI9 | MGAP_HUMAN | | | | MAX gene-associated protein | MGA, KIAA0518, MAD5 | 3,065 AA | | 336,159 Da | -4,25 | | ↓ | |
| I3L2C7 | I3L2C7_HUMAN | | | | Gem nuclear organelle associated protein 4 | GEMIN4 | 1,047 AA | | 118,822 Da | -4,25 | | ↓ | |
| E7EPN9 | E7EPN9_HUMAN | | | | Proline rich coiled-coil 2C | PRRC2C | 2,819 AA | | 308,777 Da | -4,25 | | ↓ | |
| **Supplementary Table 2.** Abundance variation in G2(T2DMwell-DL-P) versus G5(Control). 25 up-regulated and 25 down-regulated proteins | | | | | | | | | |  |  | |  |
| **Entry** | **Entry Name UNIPROT** | | | | **Protein names** | **Gene Names** | **Length** | | **Mass** | **Score** | |  | |
| P0C0L4 | CO4A_HUMAN | | | | Complement C4-A | C4A, CO4, CPAMD2 | 1,744 AA | | 192,785 Da | 6,99 | | ↑ | |
| D6RF35 | D6RF35_HUMAN | | | | Vitamin D-binding protein | GC | 476 AA | | 53,021 Da | 6,09 | | ↑ | |
| Q8TDB0 | Q8TDB0_HUMAN | | | | Apolipoprotein A-1 A175P variant |  | 50 AA | | 5,607 Da | 5,55 | | ↑ | |
| Q9NRC6 | SPTN5_HUMAN | | | | Spectrin beta chain, non-erythrocytic 5 | SPTBN5, BSPECV, HUBSPECV, HUSPECV | 3,674 AA | | 416,750 Da | 4,95 | | ↑ | |
| C0JYZ2 | C0JYZ2_HUMAN | | | | Titin | TTN | 33,423 AA | | 3,713,712 Da | 4,75 | | ↑ | |
| Q8WXI7 | MUC16_HUMAN | | | | Mucin-16 | MUC16, CA125 | 14,507 AA | | 1,519,175 Da | 4,70 | | ↑ | |
| Q9NZJ4 | SACS_HUMAN | | | | Sacsin | SACS, DNAJC29, KIAA0730 | 4,579 AA | | 521,126 Da | 4,64 | | ↑ | |
| Q15413 | RYR3_HUMAN | | | | Ryanodine receptor 3 | RYR3, HBRR | 4,870 AA | | 552,042 Da | 4,64 | | ↑ | |
| H7BZ55 | CRCC2_HUMAN | | | | Ciliary rootlet coiled-coil protein 2 | CROCC2 | 1,653 AA | | 185,641 Da | 4,52 | | ↑ | |
| A0A0A0MQT4 | A0A0A0MQT4_HUMAN | | | | DDB1- and CUL4-associated factor 10 | DCAF10 | 386 AA | | 43,197 Da | 4,46 | | ↑ | |
| Q9NR48 | ASH1L_HUMAN | | | | Histone-lysine N-methyltransferase ASH1L | ASH1L, KIAA1420, KMT2H | 2,969 AA | | 332,790 Da | 4,39 | | ↑ | |
| Q5T4S7 | UBR4_HUMAN | | | | E3 ubiquitin-protein ligase UBR4 | UBR4, KIAA0462, KIAA1307, RBAF600 | 5,183 AA | | 573,841 Da | 4,32 | | ↑ | |
| Q7Z7G8 | VP13B_HUMAN | | | | Intermembrane lipid transfer protein VPS13B | VPS13B, CHS1, COH1, KIAA0532 | 4,022 AA | | 448,664 Da | 4,25 | | ↑ | |
| F8W9J4 | F8W9J4_HUMAN | | | | Dystonin | DST | 7,461 AA | | 847,971 Da | 4,17 | | ↑ | |
| A0A087WYV5 | A0A087WYV5_HUMAN | | | | Slit homolog 2 protein | SLIT2 | 1,439 AA | | 159,980 Da | 4,17 | | ↑ | |
| Q9HCM1 | RESF1_HUMAN | | | | Retroelement silencing factor 1 | RESF1, C12orf35, KIAA1551 | 1,747 AA | | 194,857 Da | 4,09 | | ↑ | |
| E1B2D1 | E1B2D1_HUMAN | | | | Hemoglobin alpha-1 globin chain variant | HBA1 | 100 AA | | 10,763 Da | 3,91 | | ↑ | |
| Q8IYW2 | CFA46_HUMAN | | | | Cilia- and flagella-associated protein 46 | CFAP46, C10orf123, C10orf124, C10orf92, C10orf93, TTC40 | 2,715 AA | | 303,500 Da | 3,91 | | ↑ | |
| B4DDT3 | B4DDT3_HUMAN | | | | Prothrombin |  | 471 AA | | 53,149 Da | 3,91 | | ↑ | |
| Q16821 | PPR3A_HUMAN | | | | Protein phosphatase 1 regulatory subunit 3A | PPP1R3A, PP1G | 1,122 AA | | 125,767 Da | 3,81 | | ↑ | |
| Q96BZ8 | LENG1_HUMAN | | | | Leukocyte receptor cluster member 1 | LENG1 | 264 AA | | 30,528 Da | 3,81 | | ↑ | |
| B4DPI4 | B4DPI4_HUMAN | | | | cDNA FLJ53456, highly similar to Homo sapiens solute carrier family 22 (organic anion transporter), member 8 (SLC22A8), mRNA |  | 528 AA | | 58,346 Da | 3,70 | | ↑ | |
| P35580 | MYH10_HUMAN | | | | Myosin-10 | MYH10 | 1,976 AA | | 228,999 Da | 3,70 | | ↑ | |
| Q9BRS8 | LARP6_HUMAN | | | | La-related protein 6 | LARP6 | 491 AA | | 54,737 Da | 3,70 | | ↑ | |
| Q6GMV8 | Q6GMV8_HUMAN | | | | Ig-like domain-containing protein |  | 234 AA | | 24,910 Da | 3,70 | | ↓ | |
| Q8WXI7 | MUC16_HUMAN | | | | Mucin-16 | MUC16, CA125 | 14,507 AA | | 1,519,175 Da | -6,63 | | ↓ | |
| P00739 | HPTR_HUMAN | | | | Haptoglobin-related protein | HPR | 348 AA | | 39,030 Da | -6,46 | | ↓ | |
| P02774 | VTDB_HUMAN | | | | Vitamin D-binding protein | GC | 474 AA | | 52,918 Da | -6,09 | | ↓ | |
| H6VRF8 | H6VRF8_HUMAN | | | | Keratin, type II cytoskeletal 1 | KRT1 | 644 AA | | 66,053 Da | -5,32 | | ↓ | |
| T1R7A8 | T1R7A8_HUMAN | | | | MUC5AC | MUC5AC | 3,483 AA | | 350,921 Da | -5,17 | | ↓ | |
| B4DYH4 | B4DYH4_HUMAN | | | | cDNA FLJ51571, moderately similar to Mediator of DNA damage checkpoint protein 1 |  | 1,655 AA | | 178,746 Da | -5,04 | | ↓ | |
| A0A0A0MRF6 | A0A0A0MRF6_HUMAN | | | | A-kinase anchoring protein 9 | AKAP9 | 3,931 AA | | 455,919 Da | -4,86 | | ↓ | |
| B4DMR2 | B4DMR2_HUMAN | | | | cDNA FLJ50582, highly similar to Cyclin-dependent kinase-like 3 |  | 185 AA | | 20,689 Da | -4,75 | | ↓ | |
| P13645 | K1C10_HUMAN | | | | Keratin, type I cytoskeletal 10 | KRT10, KPP | 584 AA | | 58,827 Da | -4,64 | | ↓ | |
| J3KNM6 | J3KNM6_HUMAN | | | | Arf-GAP with GTPase, ANK repeat and PH domain-containing protein 2 | AGAP2 | 1,036 AA | | 108,641 Da | -4,64 | | ↓ | |
| E9M4D4 | E9M4D4_HUMAN | | | | Hemoglobin alpha-1 globin chain | HBA1 | 100 AA | | 10,783 Da | -4,58 | | ↓ | |
| G3V0E6 | G3V0E6_HUMAN | | | | Podocalyxin like | PODXL, hCG_18687 | 324 AA | | 32,702 Da | -4,58 | | ↓ | |
| B5MCN7 | B5MCN7_HUMAN | | | | Nuclear receptor coactivator 1 | NCOA1 | 1,248 AA | | 135,621 Da | -4,58 | | ↓ | |
| P35908 | K22E_HUMAN | | | | Keratin, type II cytoskeletal 2 epidermal | KRT2, KRT2A, KRT2E | 639 AA | | 65,433 Da | -4,52 | | ↓ | |
| Q9Y5S2 | MRCKB_HUMAN | | | | Serine/threonine-protein kinase MRCK beta | CDC42BPB, KIAA1124 | 1,711 AA | | 194,315 Da | -4,52 | | ↓ | |
| Q86YZ3 | HORN_HUMAN | | | | Hornerin | HRNR, S100A18 | 2,850 AA | | 282,390 Da | -4,46 | | ↓ | |
| Q96N23 | CFA54_HUMAN | | | | Cilia- and flagella-associated protein 54 | CFAP54, C12orf55, C12orf63 | 3,096 AA | | 351,970 Da | -4,46 | | ↓ | |
| E9PFQ4 | E9PFQ4_HUMAN | | | | Pleckstrin homology like domain family B member 2 | PHLDB2 | 1,115 AA | | 125,476 Da | -4,46 | | ↓ | |
| H3BRH3 | H3BRH3_HUMAN | | | | Zinc finger CCCH-type containing 18 | ZC3H18 | 196 AA | | 21,009 Da | -4,46 | | ↓ | |
| E9PGF5 | E9PGF5_HUMAN | | | | Tensin 1 | TNS1 | 1,714 AA | | 183,224 Da | -4,39 | | ↓ | |
| Q5M775 | CYTSB_HUMAN | | | | Cytospin-B | SPECC1, CYTSB, NSP5 | 1,068 AA | | 118,585 Da | -4,39 | | ↓ | |
| Q6PII6 | Q6PII6_HUMAN | | | | TMF1 protein | TMF1 | 533 AA | | 58,382 Da | -4,25 | | ↓ | |
| Q5CZC0 | FSIP2_HUMAN | | | | Fibrous sheath-interacting protein 2 | FSIP2 | 6,907 AA | | 780,607 Da | -4,25 | | ↓ | |
| B3KRD6 | B3KRD6_HUMAN | | | | cDNA FLJ34074 fis, clone FCBBF3002925, highly similar to Mus musculus SET domain containing 5 (Setd5), mRNA |  | 1,344 AA | | 147,456 Da | -4,25 | | ↓ | |
| Q14669 | TRIPC_HUMAN | | | | E3 ubiquitin-protein ligase TRIP12 | TRIP12, KIAA0045, ULF | 1,992 AA | | 220,434 Da | -4,25 | | ↓ | |
| **Supplementary Table 3.** Abundance variation in G3(DL-P) versus G5(Control). 25 up-regulated and 25 down-regulated proteins. | | | | | | | | | |  |  | |  |
| **Entry** | | | **Entry Name UNIPROT** | **Protein names** | | **Gene Names** | **Length** | **Mass** | | **Score** |  | |  |
| P0C0L4 | | | CO4A_HUMAN | Complement C4-A | | C4A, CO4, CPAMD2 | 1,744 AA | 192,785 Da | | 6,94 | ↑ | |  |
| B4E1Z4 | | | B4E1Z4_HUMAN | Complement C2 | |  | 1,266 AA | 140,943 Da | | 6,04 | ↑ | |  |
| D6RF35 | | | D6RF35_HUMAN | Vitamin D-binding protein | | GC | 476 AA | 53,021 Da | | 5,98 | ↑ | |  |
| A0A0A0MS51 | | | A0A0A0MS51_HUMAN | Gelsolin | | GSN | 748 AA | 82,526 Da | | 5,32 | ↑ | |  |
| C0JYZ2 | | | C0JYZ2_HUMAN | Titin | | TTN | 33,423 AA | 3,713,712 Da | | 5,32 | ↑ | |  |
| Q8TDB0 | | | Q8TDB0_HUMAN | Apolipoprotein A-1 A175P variant | |  | 50 AA | 5,607 Da | | 5,29 | ↑ | |  |
| H7BZ55 | | | CRCC2_HUMAN | Ciliary rootlet coiled-coil protein 2 | | CROCC2 | 1,653 AA | 185,641 Da | | 4,70 | ↑ | |  |
| Q7Z7G8 | | | VP13B_HUMAN | Intermembrane lipid transfer protein VPS13B | | VPS13B, CHS1, COH1, KIAA0532 | 4,022 AA | 448,664 Da | | 4,52 | ↑ | |  |
| Q9HCM1 | | | RESF1_HUMAN | Retroelement silencing factor 1 | | RESF1, C12orf35, KIAA1551 | 1,747 AA | 194,857 Da | | 4,39 | ↑ | |  |
| B4DK21 | | | B4DK21_HUMAN | cDNA FLJ58726 | |  | 918 AA | 102,668 Da | | 4,32 | ↑ | |  |
| B4DYT0 | | | B4DYT0_HUMAN | cDNA FLJ51150, highly similar to Sortilin-related receptor | |  | 1,124 AA | 125,786 Da | | 4,17 | ↑ | |  |
| Q68DA7 | | | FMN1_HUMAN | Formin-1 | | FMN1, FMN, LD | 1,419 AA | 157,578 Da | | 4,09 | ↑ | |  |
| B4DDT3 | | | B4DDT3_HUMAN | Prothrombin | |  | 471 AA | 53,149 Da | | 4,09 | ↑ | |  |
| Q8NF91 | | | SYNE1_HUMAN | Nesprin-1 | | SYNE1, C6orf98, KIAA0796, KIAA1262, KIAA1756, MYNE1 | 8,797 AA | 1,011,086 Da | | 4,00 | ↑ | |  |
| Q6GMX3 | | | Q6GMX3_HUMAN | IGL@ protein | | IGL@ | 236 AA | 24,700 Da | | 4,00 | ↑ | |  |
| F2Z2E2 | | | F2Z2E2_HUMAN | IQ motif containing GTPase activating protein 3 | | IQGAP3 | 1,588 AA | 179,484 Da | | 4,00 | ↑ | |  |
| G3V5X4 | | | G3V5X4_HUMAN | Spectrin repeat containing nuclear envelope protein 2 | | SYNE2 | 6,818 AA | 787,723 Da | | 4,00 | ↑ | |  |
| A0A087WV90 | | | A0A087WV90_HUMAN | Dystrophin | | DMD | 2,341 AA | 271,068 Da | | 3,91 | ↑ | |  |
| Q4G0R1 | | | Q4G0R1_HUMAN | PIBF1 protein | | PIBF1 | 698 AA | 83,111 Da | | 3,91 | ↑ | |  |
| Q2M1Z1 | | | Q2M1Z1_HUMAN | MutL homolog 3 (E. coli) | | MLH3, hCG_20493 | 1,429 AA | 161,038 Da | | 3,91 | ↑ | |  |
| A6NIW2 | | | A6NIW2_HUMAN | Dedicator of cytokinesis 11 | | DOCK11 | 2,077 AA | 238,098 Da | | 3,91 | ↑ | |  |
| H9KV90 | | | H9KV90_HUMAN | SH3 and multiple ankyrin repeat domains protein 1 | | SHANK1 | 2,169 AA | 225,900 Da | | 3,81 | ↑ | |  |
| B4E1N5 | | | B4E1N5_HUMAN | cDNA FLJ53694, highly similar to Apolipoprotein-L1 | |  | 339 AA | 37,859 Da | | 3,81 | ↑ | |  |
| Q8IWI9 | | | MGAP_HUMAN | MAX gene-associated protein | | MGA, KIAA0518, MAD5 | 3,065 AA | 336,159 Da | | 3,81 | ↑ | |  |
| B7Z326 | | | B7Z326_HUMAN | SLAIN motif family member 1 | | SLAIN1 | 304 AA | 33,498 Da | | 3,81 | ↓ | |  |
| P00739 | | | HPTR_HUMAN | Haptoglobin-related protein | | HPR | 348 AA | 39,030 Da | | -6,46 | ↓ | |  |
| P02774 | | | VTDB_HUMAN | Vitamin D-binding protein | | GC | 474 AA | 52,918 Da | | -6,09 | ↓ | |  |
| P00751 | | | CFAB_HUMAN | Complement factor B | | CFB, BF, BFD | 764 AA | 85,533 Da | | -5,93 | ↓ | |  |
| A7Y9J9 | | | A7Y9J9_HUMAN | Mucin 5AC, oligomeric mucus/gel-forming | | MUC5AC | 6,207 AA | 648,803 Da | | -5,55 | ↓ | |  |
| H6VRF8 | | | H6VRF8_HUMAN | Keratin, type II cytoskeletal 1 | | KRT1 | 644 AA | 66,053 Da | | -5,32 | ↓ | |  |
| B2R950 | | | B2R950_HUMAN | cDNA, FLJ94213, highly similar to Homo sapiens pregnancy-zone protein (PZP), mRNA | |  | 1,482 AA | 163,871 Da | | -5,21 | ↓ | |  |
| T1R7A8 | | | T1R7A8_HUMAN | MUC5AC | | MUC5AC | 3,483 AA | 350,921 Da | | -5,17 | ↓ | |  |
| B7Z992 | | | B7Z992_HUMAN | Gelsolin | |  | 715 AA | 78,832 Da | | -5,13 | ↓ | |  |
| A0A0A0MRF6 | | | A0A0A0MRF6_HUMAN | A-kinase anchoring protein 9 | | AKAP9 | 3,931 AA | 455,919 Da | | -4,86 | ↓ | |  |
| Q6P4R8 | | | NFRKB_HUMAN | Nuclear factor related to kappa-B-binding protein | | NFRKB, INO80G | 1,299 AA | 139,001 Da | | -4,75 | ↓ | |  |
| Q9UKN1 | | | MUC12_HUMAN | Mucin-12 | | MUC12, MUC11 | 5,478 AA | 558,164 Da | | -4,75 | ↓ | |  |
| B4DMR2 | | | B4DMR2_HUMAN | cDNA FLJ50582, highly similar to Cyclin-dependent kinase-like 3 | |  | 185 AA | 20,689 Da | | -4,75 | ↓ | |  |
| P13645 | | | K1C10_HUMAN | Keratin, type I cytoskeletal 10 | | KRT10, KPP | 584 AA | 58,827 Da | | -4,64 | ↓ | |  |
| J3KNM6 | | | J3KNM6_HUMAN | Arf-GAP with GTPase, ANK repeat and PH domain-containing protein 2 | | AGAP2 | 1,036 AA | 108,641 Da | | -4,64 | ↓ | |  |
| Q12955 | | | ANK3_HUMAN | Ankyrin-3 | | ANK3 | 4,377 AA | 480,410 Da | | -4,58 | ↓ | |  |
| G3V0E6 | | | G3V0E6_HUMAN | Podocalyxin like | | PODXL, hCG_18687 | 324 AA | 32,702 Da | | -4,58 | ↓ | |  |
| B5MCN7 | | | B5MCN7_HUMAN | Nuclear receptor coactivator 1 | | NCOA1 | 1,248 AA | 135,621 Da | | -4,58 | ↓ | |  |
| D6RFH5 | | | D6RFH5_HUMAN | Folliculin interacting protein 2 | | FNIP2 | 673 AA | 74,715 Da | | -4,52 | ↓ | |  |
| P35908 | | | K22E_HUMAN | Keratin, type II cytoskeletal 2 epidermal | | KRT2, KRT2A, KRT2E | 639 AA | 65,433 Da | | -4,52 | ↓ | |  |
| Q9Y5S2 | | | MRCKB_HUMAN | Serine/threonine-protein kinase MRCK beta | | CDC42BPB, KIAA1124 | 1,711 AA | 194,315 Da | | -4,52 | ↓ | |  |
| O60382 | | | O60382_HUMAN | KIAA0324 | | KIAA0324 | 1,791 AA | 191,307 Da | | -4,46 | ↓ | |  |
| Q86YZ3 | | | HORN_HUMAN | Hornerin | | HRNR, S100A18 | 2,850 AA | 282,390 Da | | -4,46 | ↓ | |  |
| Q96N23 | | | CFA54_HUMAN | Cilia- and flagella-associated protein 54 | | CFAP54, C12orf55, C12orf63 | 3,096 AA | 351,970 Da | | -4,46 | ↓ | |  |
| E9PFQ4 | | | E9PFQ4_HUMAN | Pleckstrin homology like domain family B member 2 | | PHLDB2 | 1,115 AA | 125,476 Da | | -4,46 | ↓ | |  |
| H3BRH3 | | | H3BRH3_HUMAN | Zinc finger CCCH-type containing 18 | | ZC3H18 | 196 AA | 21,009 Da | | -4,46 | ↓ | |  |
| **Supplementary Table 4.** Abundance variation in G4(Perio) versus G5(Control). 25 up-regulated and 25 down-regulated proteins | | | | | | | | | |  |  | |  |
| **Entry** | | **Entry Name UNIPROT** | | **Protein names** | | **Gene Names** | **Length** | **Mass** | | **Score** |  | |  |
| Q8WXI7 | | MUC16_HUMAN | | Mucin-16 | | MUC16, CA125 | 14,507 AA | 1,519,175 Da | | 7,09 | ↑ | |  |
| E9PFZ2 | | E9PFZ2_HUMAN | | Ceruloplasmin | | CP | 946 AA | 108,820 Da | | 7,00 | ↑ | |  |
| B4E1Z4 | | B4E1Z4_HUMAN | | Complement C2 | |  | 1,266 AA | 140,943 Da | | 6,29 | ↑ | |  |
| T1R7Q9 | | T1R7Q9_HUMAN | | MUC5AC | | MUC5AC | 4,066 AA | 413,830 Da | | 5,58 | ↑ | |  |
| Q9UQ35 | | SRRM2_HUMAN | | Serine/arginine repetitive matrix protein 2 | | SRRM2, KIAA0324, SRL300, SRM300, HSPC075 | 2,752 AA | 299,615 Da | | 5,46 | ↑ | |  |
| Q9HCM1 | | RESF1_HUMAN | | Retroelement silencing factor 1 | | RESF1, C12orf35, KIAA1551 | 1,747 AA | 194,857 Da | | 5,21 | ↑ | |  |
| Q14676 | | MDC1_HUMAN | | Mediator of DNA damage checkpoint protein 1 | | MDC1, KIAA0170, NFBD1 | 2,089 AA | 226,666 Da | | 5,17 | ↑ | |  |
| Q9NRC6 | | SPTN5_HUMAN | | Spectrin beta chain, non-erythrocytic 5 | | SPTBN5, BSPECV, HUBSPECV, HUSPECV | 3,674 AA | 416,750 Da | | 5,00 | ↑ | |  |
| A8K5T0 | | A8K5T0_HUMAN | | Complement factor H | |  | 1,231 AA | 138,950 Da | | 5,00 | ↑ | |  |
| O15021 | | MAST4_HUMAN | | Microtubule-associated serine/threonine-protein kinase 4 | | MAST4, KIAA0303 | 2,623 AA | 284,097 Da | | 5,00 | ↑ | |  |
| B4DET1 | | B4DET1_HUMAN | | DNA-directed RNA polymerase | |  | 855 AA | 91,810 Da | | 5,00 | ↑ | |  |
| Q9NXV6 | | CARF_HUMAN | | CDKN2A-interacting protein | | CDKN2AIP, CARF | 580 AA | 61,125 Da | | 4,95 | ↑ | |  |
| Q2LD37 | | BLTP1_HUMAN | | Bridge-like lipid transfer protein family member 1 | | BLTP1, FSA, KIAA1109, KIAA1371, TWEEK | 5,005 AA | 555,482 Da | | 4,91 | ↑ | |  |
| Q99490 | | AGAP2_HUMAN | | Arf-GAP with GTPase, ANK repeat and PH domain-containing protein 2 | | AGAP2, CENTG1, KIAA0167 | 1,192 AA | 124,591 Da | | 4,91 | ↑ | |  |
| H7BZ55 | | CRCC2_HUMAN | | Ciliary rootlet coiled-coil protein 2 | | CROCC2 | 1,653 AA | 185,641 Da | | 4,75 | ↑ | |  |
| O15265 | | ATX7_HUMAN | | Ataxin-7 | | ATXN7, SCA7 | 892 AA | 95,451 Da | | 4,70 | ↑ | |  |
| Q9NR48 | | ASH1L_HUMAN | | Histone-lysine N-methyltransferase ASH1L | | ASH1L, KIAA1420, KMT2H | 2,969 AA | 332,790 Da | | 4,70 | ↑ | |  |
| Q9UF83 | | YM012_HUMAN | | Uncharacterized protein DKFZp434B061 | |  | 564 AA | 59,412 Da | | 4,70 | ↑ | |  |
| Q6F5E8 | | CARL2_HUMAN | | Capping protein, Arp2/3 and myosin-I linker protein 2 | | CARMIL2, LRRC16C, RLTPR | 1,435 AA | 154,689 Da | | 4,70 | ↑ | |  |
| F5H7V9 | | F5H7V9_HUMAN | | Tenascin | | TNC | 1,838 AA | 201,143 Da | | 4,64 | ↑ | |  |
| Q15413 | | RYR3_HUMAN | | Ryanodine receptor 3 | | RYR3, HBRR | 4,870 AA | 552,042 Da | | 4,58 | ↑ | |  |
| B4DDT3 | | B4DDT3_HUMAN | | Prothrombin | |  | 471 AA | 53,149 Da | | 4,58 | ↑ | |  |
| H3BTR6 | | H3BTR6_HUMAN | | RNA binding protein with serine rich domain 1 | | RNPS1 | 127 AA | 13,000 Da | | 4,58 | ↑ | |  |
| Q7Z7G8 | | VP13B_HUMAN | | Intermembrane lipid transfer protein VPS13B | | VPS13B, CHS1, COH1, KIAA0532 | 4,022 AA | 448,664 Da | | 4,52 | ↑ | |  |
| B4DHB8 | | B4DHB8_HUMAN | | Poly(A) polymerase | |  | 740 AA | 82,354 Da | | 4,52 | ↓ | |  |
| Q8WXI7 | | MUC16_HUMAN | | Mucin-16 | | MUC16, CA125 | 14,507 AA | 1,519,175 Da | | -6,63 | ↓ | |  |
| P00751 | | CFAB_HUMAN | | Complement factor B | | CFB, BF, BFD | 764 AA | 85,533 Da | | -5,93 | ↓ | |  |
| T1R7A8 | | T1R7A8_HUMAN | | MUC5AC | | MUC5AC | 3,483 AA | 350,921 Da | | -5,17 | ↓ | |  |
| B4DYH4 | | B4DYH4_HUMAN | | cDNA FLJ51571, moderately similar to Mediator of DNA damage checkpoint protein 1 | |  | 1,655 AA | 178,746 Da | | -5,04 | ↓ | |  |
| Q9UKN1 | | MUC12_HUMAN | | Mucin-12 | | MUC12, MUC11 | 5,478 AA | 558,164 Da | | -4,75 | ↓ | |  |
| B4DMR2 | | B4DMR2_HUMAN | | cDNA FLJ50582, highly similar to Cyclin-dependent kinase-like 3 | |  | 185 AA | 20,689 Da | | -4,75 | ↓ | |  |
| P13645 | | K1C10_HUMAN | | Keratin, type I cytoskeletal 10 | | KRT10, KPP | 584 AA | 58,827 Da | | -4,64 | ↓ | |  |
| J3KNM6 | | J3KNM6_HUMAN | | Arf-GAP with GTPase, ANK repeat and PH domain-containing protein 2 | | AGAP2 | 1,036 AA | 108,641 Da | | -4,64 | ↓ | |  |
| G3V0E6 | | G3V0E6_HUMAN | | Podocalyxin like | | PODXL, hCG_18687 | 324 AA | 32,702 Da | | -4,58 | ↓ | |  |
| B5MCN7 | | B5MCN7_HUMAN | | Nuclear receptor coactivator 1 | | NCOA1 | 1,248 AA | 135,621 Da | | -4,58 | ↓ | |  |
| P35908 | | K22E_HUMAN | | Keratin, type II cytoskeletal 2 epidermal | | KRT2, KRT2A, KRT2E | 639 AA | 65,433 Da | | -4,52 | ↓ | |  |
| Q9Y5S2 | | MRCKB_HUMAN | | Serine/threonine-protein kinase MRCK beta | | CDC42BPB, KIAA1124 | 1,711 AA | 194,315 Da | | -4,52 | ↓ | |  |
| O60382 | | O60382_HUMAN | | KIAA0324 | | KIAA0324 | 1,791 AA | 191,307 Da | | -4,46 | ↓ | |  |
| Q96N23 | | CFA54_HUMAN | | Cilia- and flagella-associated protein 54 | | CFAP54, C12orf55, C12orf63 | 3,096 AA | 351,970 Da | | -4,46 | ↓ | |  |
| E9PFQ4 | | E9PFQ4_HUMAN | | Pleckstrin homology like domain family B member 2 | | PHLDB2 | 1,115 AA | 125,476 Da | | -4,46 | ↓ | |  |
| H3BRH3 | | H3BRH3_HUMAN | | Zinc finger CCCH-type containing 18 | | ZC3H18 | 196 AA | 21,009 Da | | -4,46 | ↓ | |  |
| Q5M775 | | CYTSB_HUMAN | | Cytospin-B | | SPECC1, CYTSB, NSP5 | 1,068 AA | 118,585 Da | | -4,39 | ↓ | |  |
| B3KRD6 | | B3KRD6_HUMAN | | cDNA FLJ34074 fis, clone FCBBF3002925, highly similar to Mus musculus SET domain containing 5 (Setd5), mRNA | |  | 1,344 AA | 147,456 Da | | -4,25 | ↓ | |  |
| Q14669 | | TRIPC_HUMAN | | E3 ubiquitin-protein ligase TRIP12 | | TRIP12, KIAA0045, ULF | 1,992 AA | 220,434 Da | | -4,25 | ↓ | |  |
| Q8IWI9 | | MGAP_HUMAN | | MAX gene-associated protein | | MGA, KIAA0518, MAD5 | 3,065 AA | 336,159 Da | | -4,25 | ↓ | |  |
| I3L2C7 | | I3L2C7_HUMAN | | Gem nuclear organelle associated protein 4 | | GEMIN4 | 1,047 AA | 118,822 Da | | -4,25 | ↓ | |  |
| E7EPN9 | | E7EPN9_HUMAN | | Proline rich coiled-coil 2C | | PRRC2C | 2,819 AA | 308,777 Da | | -4,25 | ↓ | |  |
| B3KTW3 | | B3KTW3_HUMAN | | cDNA FLJ38861 fis, clone MESAN2011984, highly similar to Homo sapiens collaborates/cooperates with ARF (alternate reading frame) protein (CARF), mRNA | |  | 325 AA | 34,084 Da | | -4,25 | ↓ | |  |
| Q69EZ8 | | Q69EZ8_HUMAN | | Prothrombin | |  | 295 AA | 33,638 Da | | -4,17 | ↓ | |  |
| Q02505 | | MUC3A_HUMAN | | Mucin-3A | | MUC3A, MUC3 | 3,323 AA | 345,127 Da | | -4,17 | ↓ | |  |

**Supplementary Table 5.** Plasmatic proteins exclusive for each group. Quantitative protein values are PSM (spectrum counts).

| **G1(T2DMpoorly-DL-P)** | | | | |
| --- | --- | --- | --- | --- |
| **Accession** | **Description** | **Protein Symbol** | **UniProt Entry Name** | **PSMs** |
| D3DRR6 | Inter-alpha (Globulin) inhibitor H2, isoform CRA_a | ITIH2 | D3DRR6_HUMAN | 74 |
| Q6N092 | Putative uncharacterized protein DKFZp686K18196 (Fragment) | DKFZp686K18196 | Q6N092_HUMAN | 67 |
| A4D1A8 | Similar to Piccolo protein (Aczonin) | LOC392742 | A4D1A8_HUMAN | 26 |
| B4E1C2 | Kininogen 1, isoform CRA_b | KNG1 | B4E1C2_HUMAN | 26 |
| A0A087X055 | Ras-responsive element-binding protein 1 | RREB1 | A0A087X055_HUMAN | 22 |
| E9PCK9 | Muscular LMNA-interacting protein (Fragment) | MLIP | E9PCK9_HUMAN | 20 |
| Q5T481 | RNA-binding protein 20 | RBM20 | RBM20_HUMAN | 18 |
| H0YMD1 | Low-density lipoprotein receptor | LDLR | H0YMD1_HUMAN | 17 |
| O75095 | Multiple epidermal growth factor-like domains protein 6 | MEGF6 | MEGF6_HUMAN | 17 |
| Q6ZMT4 | Lysine-specific demethylase 7A | KDM7A | KDM7A_HUMAN | 16 |
| **G2(T2DMwell-DL-P)** | | | | |
| **Accession** | **Description** | **Protein Symbol** | **UniProt Entry Name** | **PSMs** |
| A0A0A0MQT4 | DDB1- and CUL4-associated factor 10 | DCAF10 | A0A0A0MQT4_HUMAN | 21 |
| Q5T4S7 | E3 ubiquitin-protein ligase UBR4 | UBR4 | UBR4_HUMAN | 19 |
| F8W9J4 | Dystonin | DST | F8W9J4_HUMAN | 17 |
| A0A087WYV5 | Slit homolog 2 protein | SLIT2 | A0A087WYV5_HUMAN | 17 |
| E1B2D1 | Hemoglobin alpha-1 globin chain variant (Fragment) | HBA1 | E1B2D1_HUMAN | 14 |
| Q8IYW2 | Cilia- and flagella-associated protein 46 | CFAP46 | CFA46_HUMAN | 14 |
| Q16821 | Protein phosphatase 1 regulatory subunit 3A | PPP1R3A | PPR3A_HUMAN | 13 |
| Q96BZ8 | Leukocyte receptor cluster member 1 | LENG1 | LENG1_HUMAN | 13 |
| A0A087X0N9 | Mucin-3A | MUC3A | A0A087X0N9_HUMAN | 12 |
| B4DPI4 | cDNA FLJ53456, highly similar to Homo sapiens solute carrier family 22 (organic anion transporter), member 8 (SLC22A8), mRNA |  | B4DPI4_HUMAN | 12 |
| **G3(DL-P)** | | | | |
| **Accession** | **Description** | **Protein Symbol** | **UniProt Entry Name** | **PSMs** |
| B4DK21 | cDNA FLJ58726 |  | B4DK21_HUMAN | 19 |
| B4DYT0 | cDNA FLJ51150, highly similar to Sortilin-related receptor |  | B4DYT0_HUMAN | 17 |
| Q8NF91 | Nesprin-1 | SYNE1 | SYNE1_HUMAN | 15 |
| Q6GMX3 | IGL@ protein | IGL@ | Q6GMX3_HUMAN | 15 |
| F2Z2E2 | Ras GTPase-activating-like protein IQGAP3 | IQGAP3 | F2Z2E2_HUMAN | 15 |
| Q2M1Z1 | MutL homolog 3 (E. coli) | MLH3 | Q2M1Z1_HUMAN | 14 |
| A6NIW2 | Dedicator of cytokinesis protein 11 | DOCK11 | A6NIW2_HUMAN | 14 |
| B4E1N5 | cDNA FLJ53694, highly similar to Apolipoprotein-L1 |  | B4E1N5_HUMAN | 13 |
| P13671 | Complement component C6 | C6 | CO6_HUMAN | 12 |
| Q2Y0W8 | Electroneutral sodium bicarbonate exchanger 1 | SLC4A8 | S4A8_HUMAN | 12 |
| **G4(Perio)** | | | | |
| **Accession** | **Description** | **Protein Symbol** | **UniProt Entry Name** | **PSMs** |
| E9PFZ2 | Ceruloplasmin | CP | E9PFZ2_HUMAN | 127 |
| T1R7Q9 | MUC5AC (Fragment) | MUC5AC | T1R7Q9_HUMAN | 47 |
| Q9UQ35 | Serine/arginine repetitive matrix protein 2 | SRRM2 | SRRM2_HUMAN | 43 |
| Q14676 | Mediator of DNA damage checkpoint protein 1 | MDC1 | MDC1_HUMAN | 35 |
| A8K5T0 | cDNA FLJ75416, highly similar to Homo sapiens complement factor H (CFH), mRNA |  | A8K5T0_HUMAN | 31 |
| E7EWQ5 | Microtubule-associated serine/threonine-protein kinase 4 | MAST4 | E7EWQ5_HUMAN | 31 |
| B4DET1 | cDNA FLJ53860, highly similar to DNA-directed RNA polymerase II largest subunit (EC 2.7.7.6) |  | B4DET1_HUMAN | 31 |
| Q9NXV6 | CDKN2A-interacting protein | CDKN2AIP | CARF_HUMAN | 30 |
| F8VVT9 | Arf-GAP with GTPase, ANK repeat and PH domain-containing protein 2 | AGAP2 | F8VVT9_HUMAN | 29 |
| Q9UF83 | Uncharacterized protein DKFZp434B061 |  | YM012_HUMAN | 25 |
| **G5(Control)** | | | | |
| **Accession** | **Description** | **Protein Symbol** | **UniProt Entry Name** | **PSMs** |
| P13645 | Keratin, type I cytoskeletal 10 | KRT10 | K1C10_HUMAN | 24 |
| P35527 | Keratin, type I cytoskeletal 9 | KRT9 | K1C9_HUMAN | 13 |
| B3KRD6 | cDNA FLJ34074 fis, clone FCBBF3002925, highly similar to Mus musculus SET domain containing 5 (Setd5), mRNA |  | B3KRD6_HUMAN | 18 |
| Q14669 | E3 ubiquitin-protein ligase TRIP12 | TRIP12 | TRIPC_HUMAN | 18 |
| A0A087X1N7 | Nebulin | NEB | A0A087X1N7_HUMAN | 27 |
| E9PAV3 | Nascent polypeptide-associated complex subunit alpha, muscle-specific form | NACA | NACAM_HUMAN | 17 |
| T1R7A8 | MUC5AC (Fragment) | MUC5AC | T1R7A8_HUMAN | 35 |
| Q96N23 | Cilia- and flagella-associated protein 54 | CFAP54 | CFA54_HUMAN | 21 |
| E9PFQ4 | Pleckstrin homology-like domain family B member 2 (Fragment) | PHLDB2 | E9PFQ4_HUMAN | 21 |
| Q7Z407 | CUB and sushi domain-containing protein 3 | CSMD3 | CSMD3_HUMAN | 10 |

**Supplementary Table 6.** Plasmatic proteins in common in all five groups. Quantitative protein values are PSM (spectrum counts).

|  | **Description** | **Protein Symbol** | **G1(T2DMpoorly-DL-P)** | **G1(T2DMwell-DL-P)** | **G3(DL-P)** | **G4(Perio)** | **G5(Control)** |
| --- | --- | --- | --- | --- | --- | --- | --- |
| 01 | Alpha-1-antitrypsin | SERPINA1 | 511 | 570 | 739 | 657 | 546 |
| 02 | Complement C3 | C3 | 408 | 458 | 400 | 456 | 392 |
| 03 | Apolipoprotein A-I | APOA1 | 298 | 336 | 443 | 360 | 323 |
| 04 | Apolipoprotein B (Including Ag(X) antigen) | APOB | 249 | 218 | 263 | 216 | 224 |
| 05 | Alpha-2-macroglobulin | A2M | 248 | 217 | 254 | 280 | 215 |
| 06 | Fibrinogen alpha chain | FGA | 167 | 90 | 86 | 125 | 106 |
| 07 | Haptoglobin | HP | 159 | 182 | 175 | 178 | 163 |
| 08 | Apolipoprotein A-II | APOA2 | 119 | 103 | 125 | 121 | 105 |
| 09 | Complement C4-B | C4B | 110 | 132 | 124 | 143 | 148 |
| 10 | cDNA FLJ76826, highly similar to Homo sapiens ceruloplasmin (ferroxidase) (CP), mRNA |  | 107 | 102 | 98 | 143 | 123 |
| 11 | Hemopexin | HPX | 97 | 91 | 93 | 112 | 86 |
| 12 | Alpha-1-antichymotrypsin | SERPINA3 | 76 | 71 | 72 | 114 | 74 |
| 13 | Inter-alpha (Globulin) inhibitor H4 (Plasma Kallikrein-sensitive glycoprotein) | ITIH4 | 75 | 79 | 80 | 80 | 80 |
| 14 | Fibrinogen beta chain | FGB | 74 | 65 | 68 | 76 | 65 |
| 15 | Apolipoprotein A-IV | APOA4 | 68 | 55 | 65 | 67 | 67 |
| 16 | Fibrinogen gamma chain | FGG | 66 | 57 | 59 | 72 | 67 |
| 17 | Alpha-1B-glycoprotein | A1BG | 59 | 55 | 60 | 62 | 69 |
| 18 | cDNA FLJ56821, highly similar to Inter-alpha-trypsin inhibitor heavy chain H1 |  | 56 | 49 | 68 | 74 | 72 |
| 19 | Hemoglobin subunit beta | HBB | 36 | 29 | 29 | 26 | 30 |
| 20 | Apolipoprotein E | APOE | 34 | 29 | 32 | 27 | 26 |
| 21 | Clusterin | CLU | 34 | 31 | 31 | 32 | 28 |
| 22 | Alpha-2-HS-glycoprotein | AHSG | 34 | 27 | 24 | 33 | 30 |
| 23 | Vitronectin | VTN | 32 | 30 | 30 | 32 | 23 |
| 24 | Apolipoprotein C-III | APOC3 | 30 | 26 | 32 | 21 | 23 |
| 25 | Microtubule-actin cross-linking factor 1, isoforms 1/2/3/5 | MACF1 | 30 | 21 | 14 | 20 | 32 |
| 26 | Plasma protease C1 inhibitor | SERPING1 | 27 | 33 | 38 | 28 | 25 |
| 27 | Alpha-2-antiplasmin | SERPINF2 | 26 | 20 | 21 | 22 | 21 |
| 28 | Anti-RhD monoclonal T125 kappa light chain |  | 25 | 30 | 23 | 30 | 29 |
| 29 | Serum paraoxonase/arylesterase 1 | PON1 | 24 | 18 | 25 | 18 | 24 |
| 30 | MUC19 variant 12 | MUC19 | 24 | 20 | 17 | 89 | 76 |
| 31 | Afamin | AFM | 22 | 12 | 17 | 15 | 13 |
| 32 | cDNA FLJ53950, highly similar to Angiotensinogen |  | 22 | 34 | 25 | 29 | 27 |
| 33 | Alpha-1-acid glycoprotein 1 | ORM1 | 22 | 26 | 25 | 30 | 29 |
| 34 | Apolipoprotein C-I (Fragment) | APOC1 | 21 | 20 | 18 | 21 | 18 |
| 35 | Heparin cofactor 2 | SERPIND1 | 19 | 17 | 15 | 16 | 17 |
| 36 | Antithrombin-III | SERPINC1 | 18 | 21 | 30 | 31 | 34 |
| 37 | Centrosomal protein 350kDa, isoform CRA_a | CEP350 | 17 | 14 | 9 | 27 | 13 |
| 38 | cDNA, FLJ94361, highly similar to Homo sapiens serine (or cysteine) proteinase inhibitor, clade A(alpha-1 antiproteinase, antitrypsin), member 6 (SERPINA6), mRNA |  | 15 | 12 | 3 | 13 | 11 |
| 39 | cDNA FLJ58778, highly similar to Plasminogen (EC 3.4.21.7) |  | 14 | 18 | 16 | 17 | 11 |
| 40 | cDNA FLJ53691, highly similar to Serotransferrin |  | 14 | 15 | 15 | 25 | 20 |
| 41 | Catenin alpha-2 | CTNNA2 | 13 | 12 | 15 | 7 | 7 |
| 42 | Formin-like protein 2 | FMNL2 | 13 | 13 | 14 | 12 | 12 |
| 43 | AF4/FMR2 family member 4 | AFF4 | 13 | 13 | 13 | 17 | 13 |
| 44 | Obscurin | OBSCN | 13 | 11 | 12 | 13 | 17 |
| 45 | Complement C5 | C5 | 12 | 19 | 27 | 21 | 15 |
| 46 | cDNA FLJ51597, highly similar to C4b-binding protein alpha chain |  | 11 | 11 | 23 | 10 | 13 |
| 47 | Perilipin-4 | PLIN4 | 11 | 2 | 2 | 3 | 17 |
| 48 | Ig mu chain C region | IGHM | 10 | 6 | 9 | 16 | 6 |
| 49 | Lumican | LUM | 10 | 7 | 7 | 13 | 7 |
| 50 | Complement component 9, isoform CRA_a | C9 | 10 | 13 | 15 | 22 | 14 |
| 51 | Complement C1q subcomponent subunit B (Fragment) | C1QB | 9 | 7 | 7 | 9 | 8 |
| 52 | Unconventional myosin-XVB | MYO15B | 8 | 8 | 17 | 15 | 8 |
| 53 | Complement factor I | CFI | 8 | 8 | 8 | 16 | 11 |
| 54 | Pigment epithelium-derived factor | SERPINF1 | 7 | 6 | 5 | 5 | 5 |
| 55 | cDNA, FLJ93914, highly similar to Homo sapiens histidine-rich glycoprotein (HRG), mRNA |  | 7 | 6 | 7 | 8 | 6 |
| 56 | Spatacsin | SPG11 | 6 | 6 | 8 | 10 | 11 |
| 57 | Nucleosome-remodeling factor subunit BPTF (Fragment) | BPTF | 5 | 8 | 17 | 17 | 7 |
| 58 | cDNA FLJ56936, highly similar to Vitamin K-dependent protein S |  | 4 | 4 | 2 | 2 | 3 |
| 59 | Serum amyloid A protein |  | 4 | 5 | 6 | 5 | 3 |
| 60 | Complement C1s subcomponent | C1S | 4 | 6 | 8 | 6 | 7 |
| 61 | cDNA FLJ54228, highly similar to Leucine-rich alpha-2-glycoprotein |  | 2 | 3 | 4 | 7 | 4 |
